# Global Patterns and Future Dynamics of Four Invasive Cocklebur Species Under Climate Change: Contrasting Climatic and Anthropogenic Drivers

**DOI:** 10.3390/biology15050439

**Published:** 2026-03-07

**Authors:** Yunzhi Sang, Xuan Li, Jianghua Zheng, Zhong Liang, Liang Liu, Feifei Zhang, Ke Zhang, Jun Lin, Xuan Liu

**Affiliations:** 1 College of Geography and Remote Sensing Science, Xinjiang University, Urumqi 830046, China; 107552303774@stu.xju.edu.cn (Y.S.); 107556523284@stu.xju.edu.cn (X.L.); liuliang@stu.xju.edu.cn (L.L.); 107556523299@stu.xju.edu.cn (F.Z.); 107552303793@stu.xju.edu.cn (K.Z.); 107552203696@stu.xju.edu.cn (X.L.); 2 Center for Grassland Biological Disaster Prevention and Control of Xinjiang, Urumqi 830001, China; 18999280970@163.com; 3 Xinjiang Key Laboratory of Agricultural Biosafety, Urumqi 830091, China; 4 Xinjiang Key Laboratory of Oasis Ecology, Urumqi 830046, China

**Keywords:** biological invasion, cocklebur, MaxEnt, climate change, habitat suitability

## Abstract

Climate change, together with intensifying human activities, is reshaping global plant invasion dynamics and increasingly threatening ecosystem stability and biodiversity. Cockleburs are highly invasive weeds with strong ecological plasticity and dispersal capacity, causing widespread impacts on agricultural systems and native ecosystems. Here, we used the maximum entropy (MaxEnt) model to assess the current (2001–2020) and future (2021–2040, 2041–2060, and 2061–2080) potential distributions, key driving factors, and centroid shifts of four invasive cocklebur species—*Cyclachaena xanthiifolia* (=*Iva xanthiifolia*), *Xanthium chinense*, *Xanthium italicum*, and *Xanthium spinosum*—at the global scale under current climate conditions and three Shared Socioeconomic Pathway scenarios (SSP126, SSP245, and SSP585). Species occurrence records were integrated with climatic, topographic, and anthropogenic variables to project habitat suitability. Model performance was robust, with mean training and testing area under the receiver operating characteristic curve (AUC) values > 0.8 for all species and mean true skill statistic (TSS) values > 0.8 for three species (0.660 for *Xanthium spinosum*). Suitable habitats were jointly shaped by climatic and anthropogenic factors, although the dominant drivers differed among species. *Cyclachaena xanthiifolia* and *Xanthium spinosum* were primarily constrained by temperature and precipitation, whereas *Xanthium italicum* and *Xanthium chinense* were more strongly associated with human activity. At present, suitable habitat areas for *Cyclachaena xanthiifolia*, *Xanthium chinense*, *Xanthium italicum*, and *Xanthium spinosum* were 1196.92 × 10^4^, 358.76 × 10^4^, 888.34 × 10^4^, and 1985.14 × 10^4^ km^2^, respectively. Future projections indicated overall contractions in suitable habitat, with pronounced interspecific variation. *Xanthium chinense* showed the largest mean decline (−161.23 × 10^4^ km^2^ relative to the present), whereas *Cyclachaena xanthiifolia* experienced the smallest reduction (−53.15 × 10^4^ km^2^ on average). Centroid analyses further suggested overall shifts toward higher latitudes and elevations under warming scenarios. Despite uncertainties related to climate scenario variability and assumptions inherent in species distribution modelling, these findings provide quantitative evidence to support global invasion risk assessment and climate-adaptive management of invasive cockleburs.

## Simple Summary

Invasive plants are spreading rapidly worldwide, threatening ecosystems, agriculture, and biodiversity. Climate change and human activities contribute to this spread, but their influence differs among species. Here, we mapped the current and future potential distributions of four invasive cocklebur species worldwide and evaluated the roles of climate and human-related factors. These species mainly occur in temperate and subtropical regions of the Northern Hemisphere. Future projections suggest that suitable areas will generally shrink, although the extent of change varies among species. Climate is the dominant driver for some species, whereas human activities are more important for others. Our findings provide useful evidence for early warning and management of invasive cockleburs under future climate change.

## 1. Introduction

Global climate change is a major force reshaping biodiversity and ecosystem functioning [1,2]. Rising temperatures and shifts in precipitation regimes can alter species’ physiological tolerances and habitat suitability [3,4,5], driving widespread changes in geographic distributions across taxa worldwide [6,7]. Among the most responsive groups are invasive alien plants, which often exhibit strong ecological plasticity and high dispersal capacity [8,9]. Climate warming may facilitate their expansion beyond existing ecological barriers, enabling colonization of new regions where they compete with native species [10], disrupt ecosystem processes [11,12], and impose substantial ecological and economic costs. Over recent decades, interactions between plant invasions and climate change have intensified [13], elevating biological invasions from a regional concern to a global environmental challenge [14]. Consequently, identifying high-risk invasive species and predicting their potential spread under future climate scenarios have become central priorities in global change biology and ecological security research [15,16].

The genus *Xanthium* (Asteraceae) comprises a typical group of invasive weeds that are widely distributed across temperate and subtropical regions worldwide [17,18]. Its closely related genus *Cyclachaena* (Asteraceae) shares highly similar morphological and ecological traits [19]. Here, we focus on four invasive cocklebur species—*Cyclachaena xanthiifolia* (=*Iva xanthiifolia*), *Xanthium chinense*, *Xanthium italicum*, and *Xanthium spinosum*—all of which exhibit strong environmental adaptability and high reproductive capacity [20,21]. Their seeds can remain dormant for extended periods and disperse over long distances, facilitating spread via crop production, livestock movement, and transportation networks [22,23]. Consequently, these species have been listed as harmful invasive plants and are subject to monitoring or control programs in many countries across Asia, Europe, and North America [24,25,26,27]. They commonly invade croplands, grasslands, and natural vegetation, where they compete with crops for resources and reduce agricultural productivity [28]. In addition, their invasions disrupt native plant communities [29], threaten biodiversity [30], and can trigger cascading declines in ecosystem functioning [31,32]. Although the invasiveness of *Xanthium* and related taxa has attracted considerable ecological attention, most previous studies have focused on individual species, specific traits, or physiological and ecological mechanisms [33,34]. Systematic, comparative assessments across multiple invasive cocklebur species remain scarce, particularly with respect to quantifying interspecific differences in climatic sensitivity and reliance on human-mediated dispersal under accelerating climate change.

Addressing these gaps requires analytical frameworks that integrate species occurrence data with climatic and anthropogenic drivers across broad spatial scales. Species distribution models (SDMs) provide such a framework and are widely used to estimate species’ geographic ranges and invasion potential under current and future environmental conditions. Among these approaches, the maximum entropy (MaxEnt) model has been extensively applied to invasive plants because it performs well with presence-only data and often yields strong predictive accuracy [35,36]. By combining occurrence records with environmental predictors, SDMs can project potential habitat suitability under present-day conditions and future scenarios, thereby supporting assessments of climate change impacts on species range dynamics and expansion risk [37,38]. In recent years, many studies have incorporated future scenarios based on Representative Concentration Pathways (RCPs) or Shared Socioeconomic Pathways (SSPs), substantially improving our understanding of invasive alien plant spread under climate change [39,40]. Notably, SSPs explicitly link climatic forcing with socioeconomic development trajectories, enabling invasion risk to be evaluated as a combined outcome of climatic constraints and human activity intensity [41]. Here, we used three SSP scenarios—SSP126, SSP245, and SSP585—representing low-, intermediate-, and high-emission futures, respectively, for the 2030s, 2050s, and 2070s [42,43]. However, most existing studies focus on single species or local scales [44], and few have compared the climatic responses of multiple closely related species at the global scale. Moreover, spatiotemporal patterns of habitat centroid shifts remain insufficiently explored [45,46], even though such information is critical for assessing long-term invasion risks and ecological impacts. Therefore, multi-species, cross-regional analyses of habitat suitability and distribution dynamics are essential for clarifying the potential spread of invasive plants under a changing climate.

Building on this context, we focus on four invasive cocklebur species with broad global distributions and high invasion potential: *Cyclachaena xanthiifolia*, *Xanthium italicum*, *Xanthium chinense*, and *Xanthium spinosum*. Using the MaxEnt model, we integrated climatic, topographic, vegetation, and human-activity variables to project potential habitat dynamics and centroid shifts under current conditions and three Shared Socioeconomic Pathway (SSP) scenarios (SSP126, SSP245, and SSP585). Specifically, we aimed to (1) characterize the spatial patterns and key drivers of current global habitat suitability for the four species, (2) project future spatial patterns and spatiotemporal dynamics of suitable habitat under alternative scenarios, and (3) quantify the magnitude and direction of centroid shifts and identify potential expansion pathways. By jointly considering climatic and anthropogenic dimensions within a unified modelling framework, this study advances understanding of species-specific invasion dynamics and provides quantitative evidence to support global invasion risk assessment and targeted management under future climate change.

## 2. Materials and Methods

### 2.1. Species Distribution Data

**Figure 1:**
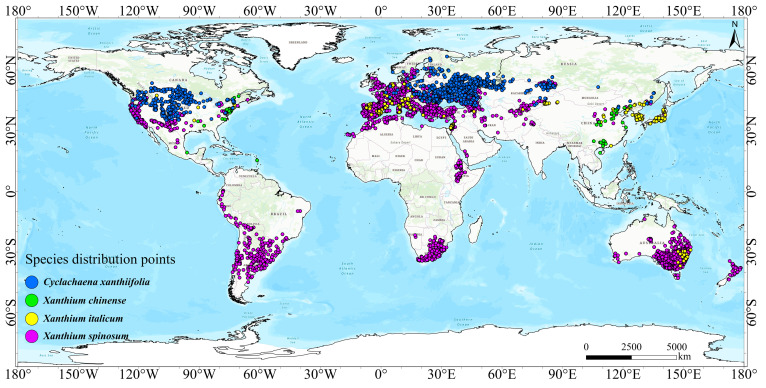
Global distribution of four invasive cocklebur species.

Occurrence records for the four invasive cocklebur species—*Cyclachaena xanthiifolia*, *Xanthium chinense*, *Xanthium italicum*, and *Xanthium spinosum*—were compiled from multiple sources. Global occurrence data were primarily obtained from the Global Biodiversity Information Facility [47,48,49,50] (GBIF; https://www.gbif.org, accessed on 17 October 2024), whereas records within China were collected from the Chinese Virtual Herbarium (CVH; https://www.cvh.ac.cn, accessed on 17 October 2024), relevant published literature, and field surveys conducted by our research team. In total, we assembled 5117 records for *Cyclachaena xanthiifolia*, 2112 for *Xanthium italicum*, 102 for *Xanthium chinense*, and 17,159 for *Xanthium spinosum*. To reduce the sensitivity of MaxEnt to spatial sampling bias, we applied spatial thinning to the occurrence records using an package in R v4.3.3. After thinning, 961 records of *Cyclachaena xanthiifolia*, 238 of *Xanthium italicum*, 54 of *Xanthium chinense*, and 2649 of *Xanthium spinosum* were retained for MaxEnt modelling (Figure 1).

### 2.2. Environmental Variables

This study used 26 environmental variables as predictors of suitable habitats for the four species. These included natural factors (topography, soil type, and bioclimatic variables) and human-related factors (population density, gross domestic product (GDP), and land use) (Table S1). The datasets and their sources are summarized in Table 1. Topographic and soil factors reflect the basic natural attributes of species habitats. Bioclimatic variables describe the spatial and temporal patterns of temperature and precipitation. Population density, GDP, and land-use type represent the intensity and characteristics of human disturbance. Integrating multidimensional information from natural and socioeconomic factors helps to reveal the mechanisms driving potential suitable habitat for the four species. Elevation data were obtained from the WorldClim v2.1 database [51]. Slope and aspect were derived using the Surface Analysis tool in ArcGIS 10.2. Soil data were sourced from the Harmonized World Soil Database (HWSD) v2.0. We extracted surface soil attributes including organic carbon content, sand content, pH, rootable depth, and total nitrogen content. Current climatic conditions were derived from monthly mean minimum temperature (°C), mean maximum temperature (°C), and total precipitation (mm) from historical climate data downloaded from WorldClim v2.1 at a spatial resolution of 30 arc-seconds. These data were then processed using the dismo package in R v4.3.3 to generate bioclimatic predictors [52]. Future climate projections were based on the BCC-CSM2-MR global climate model under three Shared Socioeconomic Pathway scenarios (SSP126, SSP245, and SSP585), also obtained from WorldClim v2.1. The spatial resolution of the future climate data was consistent with that of the historical baseline dataset. Current population density data were obtained from LandScan, and future data were provided by the National Tibetan Plateau Data Center [53]. Gross domestic product (GDP) data were obtained from the Zenodo repository [54]. Current land-use data were sourced from the ESA 300 m classification dataset, and future land-use data were taken from the Global Future Land-Use and Land-Cover (LULC) dataset [55]. To maintain temporal consistency with the climatic projections, all socioeconomic variables were aligned with the Shared Socioeconomic Pathway (SSP) scenarios (SSP126, SSP245, SSP585) and corresponding timeframes (2030s, 2050s, and 2070s). Population density, GDP, and land use were included as proxies for propagule pressure, disturbance intensity, and human-mediated dispersal [56], which are key mechanisms underlying plant invasions. To minimize the MaxEnt model’s sensitivity to sampling bias, we spatially thinned the occurrence records using the spThin package in R 4.3.3 [57], applying a minimum distance of 10 km between retained records. This threshold was chosen to reduce spatial autocorrelation and to align with the spatial resolution of the environmental predictors, as commonly adopted in global-scale species distribution modelling studies [58,59]. Finally, all layers were standardized to the WGS 1984 geographic coordinate system.

Pairwise Spearman rank correlation coefficients were calculated in R v4.3.3to reduce multicollinearity among environmental variables, and variables showing strong correlations (|r| ≥ 0.75) were removed to improve model stability [60,61]. In total, 18 variables were retained to construct the ecological niche models for the four species: altitude, slope, aspect, topsoil organic carbon, topsoil pH, topsoil sand content, topsoil root depth, GDP, land-use type, population density, mean diurnal range (bio2), isothermality (bio3), temperature seasonality (bio4), mean temperature of the wettest quarter (bio8), mean temperature of the driest quarter (bio9), precipitation of the driest month (bio14), precipitation seasonality (bio15), and mean precipitation of the warmest quarter (bio18) (Figure S1).

### 2.3. Model Construction

Prior to model implementation, the ENMeval package (2.0) was used to systematically optimize model parameters for the four target species—*Cyclachaena xanthiifolia*, *Xanthium chinense*, *Xanthium italicum*, and *Xanthium spinosum*—with the aim of reducing overfitting commonly associated with default MaxEnt settings. Parameter tuning focused on two key components: the feature combination (FC) and the regularization multiplier (RM), both of which play critical roles in balancing model complexity and predictive performance [61,62]. For FC optimization, five fundamental feature types provided by the MaxEnt framework—linear (L), quadratic (Q), hinge (H), product (P), and threshold (T)—were considered and combined into six candidate FC schemes (H, L, LQ, LQH, LQHP, and LQHPT). RM values were tested over a range of 0.5 to 5.0 at intervals of 0.5, resulting in a total of 60 distinct FC–RM parameter combinations. Based on a comprehensive evaluation of model performance across all combinations, the optimal parameter sets were identified for each species: FC = LQHP and RM = 3.5 for *Cyclachaena xanthiifolia*; FC = LQH and RM = 4.5 for *Xanthium chinense*; FC = LQ and RM = 1.0 for *Xanthium italicum*; and FC = LQHPT and RM = 4.0 for *Xanthium spinosum*.

Subsequently, MaxEnt (v3.4.4) models were implemented in a Java-based environment using the optimized parameters, together with species occurrence records and the selected environmental predictors. We randomly allocated 25% of the occurrence records for testing and used the remaining 75% for training. The model was run with 10 replicates using cross-validation to improve data utilization. The maximum number of iterations was set to 500, and 10,000 background points were randomly selected. This number has been widely adopted in large-scale species distribution modelling studies and provides a balance between model stability and computational efficiency [63,64]. All remaining parameters were retained at their default values to ensure consistent comparisons among species and to avoid overfitting associated with excessive parameter tuning [65,66]. The final ASCII output files were generated as the average across the 10 replicates.

### 2.4. Model Evaluation

The performance of the MaxEnt models was evaluated using two complementary metrics: the area under the receiver operating characteristic curve (AUC) and the true skill statistic (TSS), which together provide a robust assessment of predictive accuracy for invasive species distribution modelling.

AUC quantifies the model’s ability to discriminate between suitable and unsuitable habitats by comparing predicted suitability with observed occurrence records [35,67,68]. TSS was used to address potential imbalance in species occurrence data, as it is a threshold-dependent metric that integrates both sensitivity (true positive rate, TPR) and specificity (true negative rate, TNR) and is less sensitive to prevalence than many alternative measures [69]. The TSS formula is

TSS = TPR + TNR − 1,(1)

By combining AUC (overall discriminatory ability) and TSS (robustness under imbalanced data), we ensured comprehensive validation of model reliability, consistent with the challenges of multi-species global invasion risk assessment. The criteria for interpreting AUC [38] and TSS [70] values are provided in Table 2.

### 2.5. Habitat Suitability Classification and Centroid Shifts

To reduce the risk of omitting true species occurrences while avoiding overprediction of suitable areas, habitat suitability was classified using the maximum training sensitivity plus specificity (MTSS) threshold. The MTSS threshold was applied to the logistic output. Habitat suitability was then reclassified into four categories: unsuitable (<MTSS), slightly suitable (MTSS–0.4), moderately suitable (0.4–0.6), and highly suitable (0.6–1) [71,72]. We calculated the area of each suitability class under current conditions as well as under three Shared Socioeconomic Pathway (SSP) scenarios—SSP126, SSP245, and SSP585—for the 2030s, 2050s, and 2070s. These scenarios encompass a wide range of potential future climates and socioeconomic conditions, allowing a comprehensive evaluation of species responses under differing degrees of climatic and human influence.

Analyzing habitat centroid shifts at the global scale can reveal systematic changes in overall distribution patterns, but such results may mask substantial regional differences [73]. Because continents differ markedly in climate, topography, and the intensity of human activities, invasive species often exhibit strong spatial heterogeneity in their responses across regions. Relying solely on global centroid shifts may therefore lead to underestimation or misinterpretation of actual spread risks. Accordingly, we used the “Centroid Changes (Lines)” tool in SDMtoolbox to quantify centroid shifts for the four species across five continents under the three future scenarios. This approach captures spatiotemporal shifts in distribution centres and highlights regional variation.

## 3. Results

### 3.1. Model Performance Evaluation

Model performance metrics for the four invasive cocklebur species across different time periods are summarized in Table S2. Across all periods, mean AUC values exceeded 0.8 (Table 3), indicating strong discriminatory ability in separating occurrence records from background points and reaching “very good” to “excellent” performance based on the criteria in Table 2. Mean TSS values were >0.8 for *Cyclachaena xanthiifolia*, *Xanthium chinense*, and *Xanthium italicum* across all periods, suggesting a strong balance between sensitivity and specificity. For *Xanthium spinosum*, mean TSS values ranged from 0.660 to 0.738, which still meets the “good” performance category. Overall, the combined AUC and TSS results indicate that MaxEnt produced reliable predictions of potential habitat suitability for the four species and captured their broad distribution patterns. Notably, *Xanthium chinense* exhibited an exceptionally high AUC (0.996). Given the relatively small number of occurrence records retained after thinning (*n* = 54), this estimate should be interpreted with caution. Although model complexity was systematically tuned using ENMeval (RM = 4.5), uncertainty associated with limited sample size cannot be entirely ruled out.

### 3.2. Key Influencing Factors

**Figure 2:**
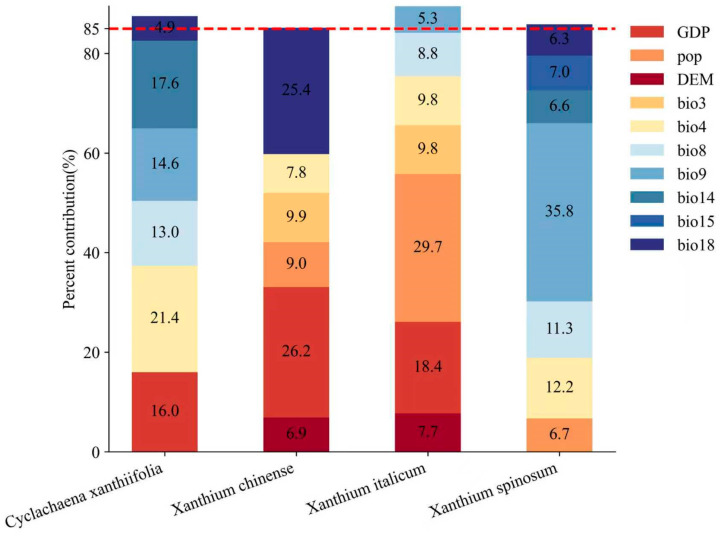
Percent contribution of each influence factor and cumulative contribution rate (the red dotted line represents a contribution rate of 85%).

In general, variables with a cumulative contribution >85% are considered the main contributors [74]. The MaxEnt results indicated that habitat suitability for the four species was jointly driven by climatic and anthropogenic factors, although the dominant contributors differed markedly among species (Figure 2). For *Cyclachaena xanthiifolia*, suitability was strongly influenced by temperature and precipitation variables. Temperature seasonality (bio4) contributed the most (21.40%), and together with precipitation of the driest month (bio14), mean temperature of the driest quarter (bio9), and mean temperature of the wettest quarter (bio8), the cumulative contribution reached 87.80%. Similarly, suitability for *Xanthium spinosum* was primarily explained by climatic variables. Mean temperature of the driest quarter (bio9) had the highest contribution (35.80%), and together with temperature seasonality (bio4), mean temperature of the wettest quarter (bio8), precipitation seasonality (bio15), and precipitation of the driest month (bio14), the cumulative contribution reached 85.90%. In contrast, suitability for *Xanthium italicum* was more strongly associated with human-related factors. Population density (29.70%) and GDP (18.40%) were the leading contributors, and together with temperature-related variables (isothermality (bio3), temperature seasonality (bio4), and mean temperature of the wettest quarter (bio8)), the cumulative contribution reached 88.0%. *Xanthium chinense* was jointly influenced by GDP, mean precipitation of the warmest quarter (bio18), and population density, with a cumulative contribution of 85.20%. Permutation importance showed patterns broadly consistent with the percent contribution rankings, and jackknife tests further supported the dominant roles of these key variables.

Overall, these results reveal clear interspecific differences in both the composition and ranking of key predictors. Climatic variables dominated the contribution structure for *Cyclachaena xanthiifolia* and *Xanthium spinosum*, whereas anthropogenic variables were more prominent for *Xanthium italicum* and *Xanthium chinense*, highlighting contrasting environmental associations of habitat suitability among the four invasive cocklebur species.

### 3.3. Current Spatial Distribution Patterns of Suitable Habitats

**Figure 3:**
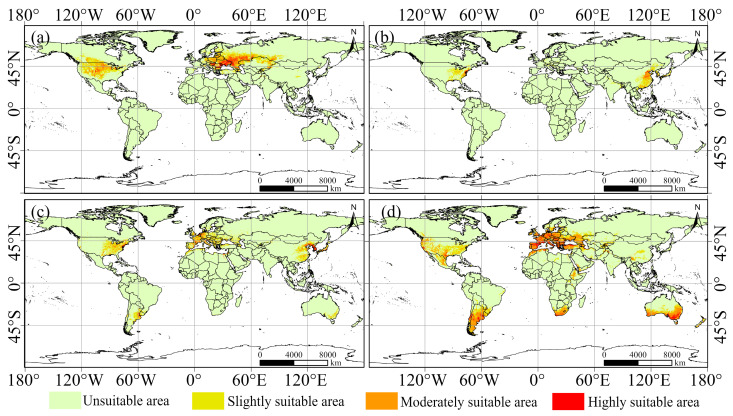
The current distribution of suitable areas for four invasive cocklebur species: (**a**) *Cyclachaena xanthiifolia*, (**b**) *Xanthium chinense*, (**c**) *Xanthium italicum*, and (**d**) *Xanthium spinosum*.

Using the MaxEnt model, we mapped suitable habitat for the four species under current climatic conditions (Figure 3) and quantified the area of each suitability class based on the classification scheme (Table 4). At present, the four species show markedly different global distribution patterns. Suitable habitat for *Cyclachaena xanthiifolia* (Figure 3a) is mainly located in central North America and from southeastern to eastern Europe, covering approximately 1196.92 × 10^4^ km^2^. Highly suitable areas are concentrated across large parts of the northern Mediterranean region. Suitable habitat for *Xanthium chinense* (Figure 3b) has the smallest extent (358.76 × 10^4^ km^2^), accounting for 2.66% of the global land area, and is primarily concentrated in two regions: eastern Asia and eastern North America. Suitable habitat for *Xanthium italicum* (Figure 3c) is mainly distributed in eastern Asia, eastern North America, Europe, South America, and southern Oceania, covering approximately 888.34 × 10^4^ km^2^. In contrast, *Xanthium spinosum* (Figure 3d) shows the broadest distribution, with suitable habitat occurring on all continents and the largest extent among the four species (1985.14 × 10^4^ km^2^), representing 14.70% of the global land area. Moderately and highly suitable classes account for a large proportion of this area (59.43% of the total suitable habitat).

### 3.4. Spatial Distribution Patterns of Suitable Habitats Under Different Climate Change Scenarios

**Figure 4:**
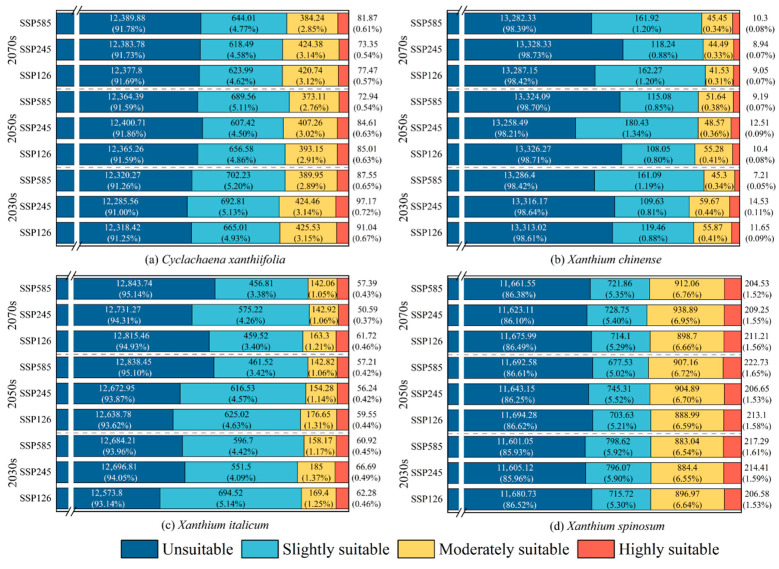
Area and proportion of the suitable areas of four invasive cocklebur species, (**a**) *Cyclachaena xanthiifolia*, (**b**) *Xanthium chinense*, (**c**) *Xanthium italicum*, and (**d**) *Xanthium spinosum*, under different climate change scenarios.

Under the SSP126, SSP245, and SSP585 scenarios, the broad suitable ranges of the four cocklebur species remained largely consistent with the present period. However, their spatiotemporal patterns and area proportions (Figure 4) changed across the 2030s, 2050s, and 2070s.

For *Cyclachaena xanthiifolia*, suitable habitat under future scenarios was mainly concentrated in mid-latitude regions, including eastern Europe, central Asia, and central North America (Figure S2). Total suitable area ranged from 1099.29 × 10^4^ to 1214.44 × 10^4^ km^2^, accounting for 8.14–9.00% of the global land area. The smallest suitable area occurred under SSP245 in the 2050s, whereas the largest occurred under SSP245 in the 2030s. For *Xanthium chinense*, suitable habitat was chiefly concentrated along the coasts of eastern China and the eastern United States (Figure S3). Total suitable area remained relatively small (171.67 × 10^4^–217.67 × 10^4^ km^2^), representing 1.27–1.79% of the global land area. The smallest suitable area occurred under SSP245 in the 2070s, while the largest occurred under SSP585 in the 2070s. A highly suitable habitat for *Xanthium chinense* was primarily located on the North China Plain and around Washington State (USA), but its extent was very limited, generally below 15 × 10^4^ km^2^, and as low as 7.21 × 10^4^ km^2^ under the SSP585 scenario in the 2030s. For *Xanthium italicum*, suitable habitat was largely distributed across southern Europe, Asia, eastern North America, South America, and parts of Australia (Figure S4). Total suitable area ranged from 656.26 × 10^4^ to 926.20 × 10^4^ km^2^ (4.86–6.86% of the global land area). The smallest area occurred under SSP585 in the 2070s, whereas the largest occurred under SSP126 in the 2030s. Highly suitable areas were mainly located in western Europe, eastern North America, and the Korean Peninsula, ranging from 50.59 × 10^4^ to 66.69 × 10^4^ km^2^ and accounting for 0.37–0.49% of the global land area. For *Xanthium spinosum*, suitable habitat remained widespread across all continents (Figure S5). Total suitable area ranged from 1805.72 × 10^4^ to 1898.95 × 10^4^ km^2^, accounting for 13.38–14.07% of the global land area and representing the largest extent among the four species. The smallest suitable area occurred under SSP126 in the 2050s, whereas the largest was observed under SSP585 in the 2030s. Across scenarios, total suitable area exhibited a decline–increase pattern over time. Highly suitable areas were mainly distributed along the Mediterranean coast, in southern South America, and in southeastern Australia. Their extent ranged from 204.53 × 10^4^ to 222.73 × 10^4^ km^2^, accounting for 1.52–1.65% of the global land area.

To compare differences among SSP scenarios, we calculated the mean suitable area across the 2030s, 2050s, and 2070s for each emission pathway. For *Cyclachaena xanthiifolia*, inter-scenario differences were minimal, with mean areas varying by only 4.35 × 10^4^ km^2^. *Xanthium chinense* showed moderate variability (11.20 × 10^4^ km^2^), whereas *Xanthium italicum* exhibited pronounced sensitivity to emission intensity, with a mean inter-scenario difference of 112.79 × 10^4^ km^2^, indicating substantial contraction under SSP585. *Xanthium spinosum* displayed intermediate variability (59.88 × 10^4^ km^2^), with slightly larger mean areas under SSP245. Overall, these comparisons indicate marked interspecific differences in sensitivity to emission pathways, with *Xanthium italicum* showing the strongest response.

### 3.5. Spatial Distribution Changes of Suitable Habitats Under Different Climate Change Scenarios

Under projected future climates, all four species showed notable changes in suitable habitat, with total suitable area generally decreasing relative to the current period. Overall, each species exhibited a mosaic of expansion and contraction, and the spatial patterns were jointly shaped by scenario and time, with pronounced interspecific differences. Among the four species, *Xanthium chinense* showed the largest reduction, with a mean decrease of 161.23 × 10^4^ km^2^ across future periods compared with the current period. In contrast, *Cyclachaena xanthiifolia* showed the smallest change, with a mean decrease of 53.15 × 10^4^ km^2^.

For *Cyclachaena xanthiifolia*, future changes (Figure S6) were characterized by expansion in northern and southern North America as well as northern and southern Europe, while contractions occurred in central North America, central Europe, and western Asia. Overall, suitable area decreased over time and under higher-emission scenarios. For *Xanthium chinense*, reductions were the most pronounced (Figure S7), with contractions mainly in eastern North America (near the Appalachian Mountains) and eastern Asia (along the Yellow and Bohai Seas). Expansions occurred primarily west of the contraction zones in Asia and along the margins of the current suitable range. For *Xanthium italicum*, future scenarios (Figure S8) showed distinct areas of both expansion and contraction. Contractions were mainly located in eastern North America, western Europe, South America, and Oceania, with the greatest reductions under the high-emission scenario (SSP585). In contrast, expansions were primarily observed in eastern Asia and central Europe. On average, suitable area for *Xanthium italicum* is projected to decline by 110.06 × 10^4^ km^2^ relative to the current period. For *Xanthium spinosum*, projected changes under the three scenarios (Figure S9) showed a complex pattern of interwoven expansions and contractions, occurring mainly along the margins of its current suitable range across continents. Overall, suitable area declined substantially, with a mean reduction of 138.20 × 10^4^ km^2^ relative to the present period.

To further quantify the differences among SSP scenarios, we calculated the mean projected change in suitable area across the 2030s, 2050s, and 2070s for each emission pathway. Clear interspecific differences in sensitivity to emission intensity were observed. *Cyclachaena xanthiifolia* exhibited minimal inter-scenario variability, with mean reductions ranging from 50.75 to 55.10 × 10^4^ km^2^ (inter-scenario difference: 4.35 × 10^4^ km^2^). *Xanthium chinense* showed moderate variability (156.37–167.57 × 10^4^ km^2^; difference: 11.20 × 10^4^ km^2^). In contrast, *Xanthium italicum* displayed pronounced sensitivity to emission pathways, with mean reductions ranging from 64.35 × 10^4^ km^2^ under SSP126 to 177.14 × 10^4^ km^2^ under SSP585 (difference: 112.79 × 10^4^ km^2^). *Xanthium spinosum* exhibited intermediate variability (difference: 59.88 × 10^4^ km^2^), with comparatively smaller reductions under SSP245. Overall, species-specific sensitivity to emission pathways followed the order *Xanthium italicum* > *Xanthium spinosum* > *Xanthium chinense* > *Cyclachaena xanthiifolia*, indicating that high-emission scenarios tend to amplify habitat contraction for more climate-sensitive species.

### 3.6. Potential Distribution Centroid Shifts of Four Species

**Figure 5:**
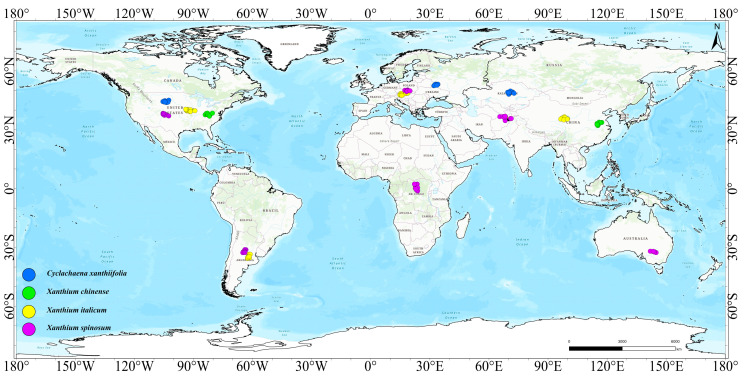
Potential distribution centers of four invasive cocklebur species.

Centroid shifts of the four invasive species across continents (Figure 5; Table S3) revealed contrasting migration patterns, with some species shifting toward higher latitudes and elevations, whereas others moved toward lower latitudes or downslope habitats.

*Cyclachaena xanthiifolia* showed a clear northward and upward shift in Asia (Figure S10a), with the centroid moving from 48.11° N and 379 m (present) to 48.59° N and 718 m under SSP585 in the 2070s. In Europe (Figure S10b), the centroid remained relatively stable with minimal displacement, whereas in North America (Figure S10c) it shifted markedly westward and upward, from 921 m to approximately 1300 m, indicating a tendency toward higher elevations and more western regions. *Xanthium chinense* exhibited a moderate latitudinal response but a stronger elevational increase. In Asia (Figure S11a), centroid elevation increased slightly from 39 m to 42 m under SSP126 by the 2070s. In North America (Figure S11b), the centroid shifted northeastward, with elevation increasing from 361 m to 841 m under SSP585 by the 2070s. However, in the 2030s and 2050s, the elevational trend was generally downward. *Xanthium italicum* showed divergent patterns among continents. In North America (Figure S12c), the centroid shifted toward higher latitudes and elevations, from 89.21° W, 39.69° N, and 183 m (present) to 92.60° W, 39.90° N, and 232 m (future mean). In contrast, in Asia (Figure S12a), Europe (Figure S12b), and South America (Figure S12d), the centroid shifted toward lower latitudes but higher elevations, suggesting a tendency toward montane environments. *Xanthium spinosum* exhibited the most heterogeneous centroid responses among continents. In Africa (Figure S13c), both latitude and elevation changed only modestly, with elevation remaining close to the present value (453 m) across future scenarios, indicating relatively stable distribution centres. In South America (Figure S13f), centroid elevation increased moderately relative to the present level of 164 m under several scenarios, with limited latitudinal displacement. In contrast, Eurasian regions showed a general downward shift in centroid elevation relative to present conditions, with Europe (Figure S13b) declining from 898 m and Asia (Figure S13a) also exhibiting reduced elevations under most projections. North America (Figure S13d) and Oceania (Figure S13e) displayed fluctuating elevational responses without a consistent directional trend, while latitudinal shifts were generally limited across continents.

## 4. Discussion

### 4.1. Key Influencing Factors of Different Species

Jackknife analysis indicated that the potential distributions of the four invasive cocklebur species are jointly shaped by climatic and anthropogenic factors, although the relative importance of these drivers varies substantially among species. These interspecific differences suggest that the studied taxa are constrained by distinct ecological filters and dispersal pathways.

For *Cyclachaena xanthiifolia* and *Xanthium spinosum*, climatic variables—particularly temperature seasonality (bio4) and mean temperature of the driest quarter (bio9)—were the most influential predictors. This pattern aligns with the expectation that distributional limits of many herbaceous invasive plants are closely linked to physiological sensitivity to thermal and moisture stress. High-temperature seasonality can disrupt phenological cues, including the timing of flowering and seed maturation, and increase exposure to adverse temperatures during vulnerable life stages such as germination and early establishment [75,76]. Likewise, the mean temperature of the driest quarter (bio9) is closely related to drought stress [77]; higher temperatures during already dry periods intensify evapotranspiration, potentially pushing plants beyond hydraulic safety margins and limiting survival and reproduction [78]. Together, these results are consistent with a climate-constrained invasion strategy in which geographic expansion is largely bounded by abiotic tolerance limits [79,80].

In contrast, the distribution of *Xanthium italicum* showed a stronger association with anthropogenic variables, particularly GDP and population density. These variables are commonly used as proxies for the intensity of human-mediated dispersal and landscape disturbance [81]. Regions with higher GDP often have denser and more interconnected transportation networks [82], which can facilitate long-distance movement of *Xanthium italicum* burs as contaminants in agricultural commodities, textiles, or livestock feed [83,84]. At the same time, economic activity can accelerate land-use change and disturbance, creating high-resource ruderal habitats that favour establishment [85]. Population density may further amplify this process by increasing the frequency of human activities that promote secondary dispersal and by sustaining a continuous supply of disturbed urban and agricultural niches [86,87]. This pattern suggests a human-facilitated strategy in which distribution is less constrained by climate and more dependent on access to human vectors and disturbed landscapes [88]. *Xanthium chinense* showed a mixed pattern, reflecting the combined influence of climatic and socioeconomic factors. Its niche was strongly associated with precipitation during the warmest quarter (bio18), a key climatic filter that determines water availability during peak growth and thus helps define its fundamental niche [89]. Within these climatic constraints, however, human activities may contribute to local spread and abundance by facilitating dispersal and creating suitable disturbed habitats [90,91]. As a result, *Xanthium chinense* may exploit human-modified environments and disperse through pathways similar to those of *Xanthium italicum*, albeit potentially less efficiently, which could lead to suitable areas that are broader than those expected from climate alone [92].

Overall, these findings are consistent with previous work indicating that climate sets broad physiological limits on invasion [93], whereas human activities shape realized spread by providing dispersal vectors and modifying habitat conditions [94,95]. Accordingly, the four species appear to occupy a continuum from climate-driven to human-driven invasion strategies. This dual influence underscores that invasion success depends not only on climatic suitability but also on opportunities for anthropogenic dispersal [96,97]. Recognizing these contrasting yet complementary drivers provides a mechanistic basis for interpreting the observed spatiotemporal dynamics of habitat suitability under climate change and offers important insights for developing species-specific prevention and management strategies.

### 4.2. Temporal–Spatial Dynamics of Suitable Habitats and Resultant Invasion Risks

At present, suitable habitat for the four invasive species is mainly concentrated in temperate and subtropical regions of the Northern Hemisphere, with relatively high suitability in North America, southern to central Europe, and eastern Asia. These high-suitability areas are typically surrounded by transitional zones of moderate and low suitability, forming a characteristic centre–periphery gradient [98,99]. This spatial pattern underscores the dependence of invasive plants on relatively stable climatic regimes and highlights the limiting roles of precipitation and temperature seasonality [100,101].

Under future scenarios, total suitable area generally contracts for all species, but the magnitude and direction of change differ markedly among taxa. *Xanthium chinense* shows the strongest reduction, accompanied by northward and upward centroid shifts, consistent with a high sensitivity to warming and moisture stress [102,103]. By contrast, *Cyclachaena xanthiifolia* exhibits the smallest contraction, which may be related to its broad environmental tolerance and reproductive plasticity [104]. *Xanthium italicum* and *Xanthium spinosum* display intermediate reductions but contrasting migration patterns: *Xanthium italicum* may maintain regional persistence partly through anthropogenic dispersal [105], whereas *Xanthium spinosum* tends to retreat in regions experiencing intensified heat and drought. Continental comparisons further suggest poleward and upslope shifts in Asia and North America, but comparatively smaller displacement in Europe and Oceania, potentially reflecting the buffering influence of oceanic climates and terrain heterogeneity [106,107]. Together, these differences indicate that suitability dynamics are shaped by both global warming and region-specific climatic and topographic constraints [108].

Importantly, these shifts are not simply geographic redistributions; they imply a spatial transfer of invasion risk. Our projections indicate increasing suitability in areas that are currently unsuitable or marginal, pointing to regions where invasion risk may rise under future climates. Potential hotspots include higher-latitude zones—such as northern parts of the European Plain, the Canadian Prairies, and northeastern China—where warming could relax thermal limitations [109,110], as well as high-elevation valleys and foothills in major mountain systems, including the Alps and Rocky Mountains, where weakening temperature constraints may permit upslope range expansion [111,112].

The ecological and socioeconomic implications of these shifts could be substantial. Poleward and upslope expansion of these noxious weeds poses risks to agricultural systems in emerging hotspots [113,114]. Invasion can reduce crop yields through competition for resources, contaminate harvests with prickly burs that lower product quality, and potentially harm livestock through toxicity or injury [115,116]. Moreover, incursions into natural ecosystems at higher elevations and latitudes—regions that often harbour unique biodiversity and include fragile habitats with limited prior disturbance—may displace native flora, alter soil properties, and reduce ecosystem stability [117]. Although contractions in some currently warm and dry regions could temporarily relieve local invasion pressure, this benefit may be offset by heightened pressure on newly exposed ecosystems at higher latitudes and elevations [113,118].

Collectively, the projected contractions and centroid shifts highlight non-uniform species responses to climate change [119]. This dynamic redistribution of suitability underscores the need for proactive and regionally differentiated management strategies, including enhanced monitoring and early detection in areas projected to experience increasing invasion risk [120,121].

### 4.3. Interspecific Divergence and Ecological Strategies

Despite their morphological similarity and shared evolutionary history, the four invasive species appear to adopt distinct ecological strategies that shape their responses to environmental change. *Cyclachaena xanthiifolia* and *Xanthium spinosum* exemplify a climate-constrained mode of expansion, in which temperature seasonality and precipitation variability are primary determinants of distribution. These taxa show generally poleward and elevational shifts under warming conditions, highlighting their sensitivity to climatic change [23]. In contrast, *Xanthium italicum* and *Xanthium chinense* are more consistent with a human-mediated invasion strategy, showing stronger associations with population density and GDP [122]. Their reliance on human activity may enable persistence—and even localized expansion—in economically developed regions, despite an overall climate-driven contraction of global suitable area.

The magnitude and direction of centroid shifts further differentiate these strategies. Climate-driven species tend to migrate more predictably toward cooler and/or wetter environments, whereas human-associated taxa may exhibit more irregular or multidirectional shifts, potentially modulated by trade routes and land-use intensity [96,120]. Such interspecific divergence likely reflects the combined effects of physiological tolerance, dispersal pathways, and socioeconomic connectivity [123]. Recognizing these contrasting strategies provides a useful conceptual framework for classifying invasive species under global change. Similar dual invasion modes have been documented for other widespread weeds, such as *Ambrosia artemisiifolia* and *Solidago canadensis* [124,125,126], supporting the broader relevance of a climate–human dichotomy across taxa.

Continental comparisons further highlight the combined influence of regional climate systems and anthropogenic processes in shaping the spatial dynamics of invasive *Xanthium* species. In Asia, variability in the monsoon system may promote northward expansion by creating favourable thermal and moisture conditions during the growing season [127]. However, concurrent intensification of aridity in continental interiors can constrain establishment in inland drylands, potentially fragmenting suitable habitat [128]. In Europe, oceanic climates can buffer environmental variability and may mitigate projected habitat loss under future scenarios [129,130], which could help explain the relatively small centroid shifts and persistence of core suitable areas in temperate regions. In North America, extensive agricultural corridors, dense transportation networks, and active trade routes provide pathways for propagule movement [131]. These anthropogenic dispersal processes may enhance persistence and facilitate spatial reorganization under warming, particularly in regions experiencing moderate climatic stress [132]. Collectively, these continent-specific processes suggest that future trajectories of invasive *Xanthium* species will be shaped not only by climatic forcing but also by the intensity and structure of human-mediated landscape connectivity.

### 4.4. Mechanistic Insights Under Future Climate Scenarios

To further elucidate the mechanisms underlying these divergent responses, we examined how different emission pathways may modulate habitat trajectories. The three SSP scenarios represent distinct combinations of climatic forcing and socioeconomic development [133], and they are associated with increasingly divergent temperature and hydrological regimes that can reshape habitat suitability [134]. Under SSP585, global mean temperature is projected to increase by more than 4 °C by 2100, accompanied by greater precipitation variability and a higher likelihood of drought–flood alternations in many regions [135]. For the more climate-driven species (*Cyclachaena xanthiifolia* and *Xanthium spinosum*), increased thermal variability and prolonged dry periods can narrow climatic niches by intensifying evapotranspiration and reducing soil moisture availability [136]. As a result, suitable habitat tends to shift toward higher latitudes and elevations where cooler and relatively stable conditions are more likely to persist.

For the more human-associated taxa (*Xanthium italicum* and *Xanthium chinense*), scenario-dependent outcomes may reflect not only climatic constraints but also the socioeconomic assumptions embedded in SSPs. Under SSP585, socioeconomic trends associated with rapid development and high resource demand could increase opportunities for long-distance dispersal, for example through intensified commodity movement and agricultural trade, whereby burs may be transported as contaminants in shipping containers, grain shipments, or other goods [137]. At the same time, intensive land use and sustained habitat disturbance can create corridors and “bridgehead” sites that facilitate establishment [138]. However, under this pathway, a key limitation may be the geographic decoupling between climatic suitability and centres of human activity: densely populated and economically developed regions may become too hot or dry to sustain viable populations [139], leading to habitat contraction despite elevated dispersal pressure.

By contrast, under SSP126, mitigated climate change can maintain broader thermal–moisture envelopes, potentially reducing climatic constraints on suitable habitats. Meanwhile, the associated socioeconomic trajectory may also reduce invasion opportunities. For example, more sustainable land-use practices can limit the availability of disturbed ruderal habitats [140], and strengthened biosecurity and altered trade patterns could further restrict human-mediated dispersal [141]. This combination is consistent with relatively stable (or only modestly shifting) suitable habitat for climate-driven species, but a more constrained spread potential for taxa that rely heavily on anthropogenic vectors. In addition, the enhanced climatic variability under SSP585 may increase establishment barriers in marginal habitats—through heightened environmental stress and altered biotic interactions—potentially contributing to stronger habitat contractions and larger centroid displacements under high-emission conditions [3,113]. Overall, these results highlight that increasing emission intensity can amplify physiological stress and habitat fragmentation, while also potentially intensifying the anthropogenic processes that facilitate dispersal for some species. This underscores the dual importance of mitigation: limiting climatic deterioration while steering socioeconomic development away from pathways that elevate future invasion risk.

### 4.5. Ecological and Management Implications

The divergent distributional patterns and mechanistic responses identified in this study indicate that future invasion trajectories of invasive cocklebur species will be shaped by the combined influence of climatic forcing and human facilitation. Our results suggest that invasion risk among closely related taxa lies along a climate–human facilitation continuum rather than being governed by a single dominant driver. Climate-driven taxa (*Cyclachaena xanthiifolia* and *Xanthium spinosum*) are expected to shift poleward and upslope along warming gradients, whereas more human-associated taxa (*Xanthium italicum* and *Xanthium chinense*) may persist or even increase in anthropogenically disturbed landscapes such as croplands, transportation corridors, and urban–rural interfaces. These contrasting responses underscore that invasion dynamics under global change cannot be interpreted through climatic suitability alone, but instead require an integrated socioecological perspective that explicitly accounts for human-mediated dispersal and landscape modification [142,143].

From a management perspective, our findings support multi-tiered, adaptive, and ecosystem-based strategies that respond to both ecological processes and socioeconomic realities [144]. Early detection and rapid response remain among the most effective approaches for limiting establishment in emerging hotspots, particularly in regions projected to experience increasing suitability under future climates, including higher latitudes and elevations [145,146]. Consistent with the prominent roles of population density and GDP for human-associated species, monitoring should prioritize high-risk environments characterized by intense human activity, such as agricultural production zones, logistics hubs, and major transportation networks where propagule pressure is likely to be high [147]. Timely eradication during the vegetative stage, before flowering and seed set, can substantially reduce long-term management costs [148,149]. For on-the-ground control, integrated approaches that combine physical, chemical, and ecological measures are essential [150]. Manual or mechanical removal at the seedling stage remains effective in sparsely infested areas [151], whereas well-timed herbicide applications can improve control efficiency in larger infestations when applied under appropriate environmental conditions [152]. In heavily degraded or abandoned lands, ecological replacement—through the establishment of competitive native species or non-invasive economic plants—may provide sustainable suppression while supporting habitat restoration [153,154].

Institutional coordination is another critical component of invasion management under climate change [155]. Strengthened biosecurity for imported agricultural products, seeds, and livestock feed can help block key propagule entry pathways [156], while coordinated efforts among agricultural, forestry, environmental, and transportation agencies can reduce regulatory gaps and improve management efficiency [157]. In parallel, public awareness and community participation can complement technical measures by improving early detection and promoting long-term stewardship [158], particularly when embedded within structured monitoring programs.

Collectively, these measures align with ecosystem-based principles for invasive species management. Integrating monitoring, adaptive control, ecological restoration, and socio-institutional cooperation provides a comprehensive pathway to mitigate invasion risks [144,159]. Overall, this study indicates that effective management of cocklebur invasions under climate change may require a shift from short-term eradication campaigns toward preventive, landscape-level management that accounts for species-specific invasion strategies and is embedded within regional climate adaptation and biodiversity conservation policies.

### 4.6. Limitation and Uncertainty

Nevertheless, several limitations and sources of uncertainty should be acknowledged. First, global occurrence records for the four species are spatially uneven, and, for taxa with relatively small sample sizes (e.g., *Xanthium chinense*), uncertainty may persist despite spatial thinning and parameter optimization; projections for this species should therefore be interpreted with appropriate caution. Second, species distribution models typically assume climatic equilibrium and do not explicitly incorporate biotic interactions, which can influence realized distributions. Third, projections under SSP scenarios are inherently uncertain due to differences among climate model structures and the socioeconomic assumptions embedded in alternative pathways. Finally, the ~10 km spatial resolution of the environmental predictors may not fully capture fine-scale habitat heterogeneity [160,161]. Future work integrating higher-resolution environmental data and multi-model climate ensembles could help refine projections and strengthen invasion risk assessments under ongoing global change.

## 5. Conclusions

This study provides a global assessment of the potential distributions and migration trajectories of four invasive cocklebur species—*Cyclachaena xanthiifolia*, *Xanthium chinense*, *Xanthium italicum*, and *Xanthium spinosum*—under current conditions and future climate scenarios. By integrating climatic, topographic, and socioeconomic variables within a MaxEnt framework, we identified the principal drivers and projected dynamics of habitat suitability across continents.

Our results indicate that invasion patterns are jointly shaped by climate and human activity, although the relative importance of these drivers varies markedly among species. *Cyclachaena xanthiifolia* and *Xanthium spinosum* are more strongly associated with climatic variability, whereas *Xanthium italicum* and *Xanthium chinense* show closer associations with anthropogenic factors such as population density and economic activity. These contrasting responses suggest species-specific invasion strategies along a climate–human facilitation continuum. At present, suitable habitats are mainly concentrated in temperate and subtropical regions of the Northern Hemisphere. Projections under SSP126, SSP245, and SSP585 consistently indicate an overall contraction of suitable habitat, accompanied by pronounced interspecific and regional differences. Centroid analyses further reveal heterogeneous migration trajectories: although poleward and upslope shifts generally prevail under warming, some regional populations shift toward lower latitudes or elevations, reflecting local climatic, topographic, and anthropogenic constraints. As thermal barriers weaken, invasion risk is likely to increase in higher-latitude and high-elevation regions.

Overall, this study advances invasion ecology by jointly considering climatic and anthropogenic dimensions within a unified predictive framework and highlights the importance of species-specific perspectives when assessing future invasion risks. These findings provide quantitative evidence to anticipate large-scale shifts in invasion pressure and support the development of adaptive, forward-looking management strategies in a rapidly changing global environment.

## Figures and Tables

**Table 1:** Primary data information and sources.

Data Type	Time	Source of Data	Resolution
Terrain	-	Resource and Environmental Science Data Platform (https://www.resdc.cn/, accessed on 25 January 2025)	1 km
Soil variables	-	Harmonized World Soil Database (HWSD) (https://gaez.fao.org/pages/hwsd, accessed on 25 January 2025)	1 km
Bioclimatic variables	-	WorldClim (https://worldclim.org, accessed on 25 January 2025)	30 s (~1 km)
Population density	Present	Landscan (https://landscan.ornl.gov/, accessed on 25 January 2025)	1 km
	2030–2070	National Tibetan Plateau Data Center (https://data.tpdc.ac.cn/, accessed on 25 January 2025)	1 km
GDP	-	Zenodo (https://zenodo.org/, accessed on 25 January 2025)	-
Land-use type	Present	LandCover300 m (https://maps.elie.ucl.ac.be/CCI/viewer/, accessed on 25 January 2025)	300 m
	2030–2070	Figshare (https://doi.org/10.6084/m9.figshare.23542860, accessed on 25 January 2025)	1 km

**Table 2:** Accuracy evaluation standard of the MaxEnt model.

Evaluation Index	Value	Standard
AUC	0–0.6	poor
	0.6–0.7	moderate
	0.7–0.8	good
	0.8–0.9	very good
	0.9–1.0	excellent
TSS	−1–0.4	poor
	0.4–0.5	fair
	0.5–0.7	good
	0.7–0.85	very good
	0.85–0.9	excellent
	0.9–1	almost perfect to perfect

**Table 3:** Mean model accuracy of four invasive cocklebur species at different time periods.

Species	Period	AUCmean	TSSmean
*Cyclachaena xanthiifolia*	Present	0.9474	0.8212
	2030s	0.9477	0.8170
	2050s	0.9494	0.8371
	2070s	0.9493	0.8415
*Xanthium chinense*	Present	0.9950	0.9376
	2030s	0.9966	0.9552
	2050s	0.9965	0.9477
	2070s	0.9964	0.9577
*Xanthium italicum*	Present	0.9794	0.8631
	2030s	0.9805	0.8639
	2050s	0.9806	0.8844
	2070s	0.9814	0.8899
*Xanthium spinosum*	Present	0.8800	0.6603
	2030s	0.8810	0.6599
	2050s	0.8815	0.6883
	2070s	0.8827	0.7342

**Table 4:** The current area of suitable habitat for four invasive cocklebur species (×10^4^ km^2^).

Species	Unsuitable Area	Slightly Suitable Area	Moderately Suitable Area	Highly Suitable Area
*Cyclachaena xanthiifolia*	12,303.08	678.18	406.71	112.03
*Xanthium chinense*	13,141.24	268.02	70.63	20.11
*Xanthium italicum*	12,611.66	618.48	202.06	67.80
*Xanthium spinosum*	11,514.86	805.33	975.93	203.88

## Data Availability

The data and materials that support the findings of this study are provided in the Supplementary Materials.

## Supplementary Materials

The following supporting information can be downloaded at https://www.mdpi.com/article/10.3390/biology15050439/s1. Figure S1: Pearson correlation analysis of various environment variables (r < 0.75); Figure S2: Predicted suitable areas of *Cyclachaena xanthiifolia* under different climate change scenarios; Figure S3: Predicted suitable areas of *Xanthium chinense* under different climate change scenarios; Figure S4: Predicted suitable areas of *Xanthium italicum* under different climate change scenarios; Figure S5: Predicted suitable areas of *Xanthium spinosum* under different climate change scenarios; Figure S6: Spatial distribution changes of *Cyclachaena xanthiifolia* under different climate change scenarios; Figure S7: Spatial distribution changes of *Xanthium chinense* under different climate change scenarios; Figure S8: Spatial distribution changes of *Xanthium italicum* under different climate change scenarios; Figure S9: Spatial distribution changes of *Xanthium spinosum* under different climate change scenarios; Figure S10: Migration trajectories of the distribution centers of *Cyclachaena xanthiifolia* in different continents (a) Asia, (b) Europe, (c) North America under different climate change scenarios; Figure S11: Migration trajectories of the distribution centers of *Xanthium chinense* in different continents (a) Asia, (b) North America under different climate change scenarios; Figure S12: Migration trajectories of the distribution centers of *Xanthium italicum* in different continents (a) Asia, (b) Europe, (c) North America, (d) South America under different climate change scenarios; Figure S13: Migration trajectories of the distribution centers of *Xanthium spinosum* in different continents (a) Asia, (b) Europe, (c) Africa, (d) North America, (e) Oceania, (f) South America under different climate change scenarios; Table S1: Initially selected environment variables; Table S2: Model accuracy of four kinds at different periods; Table S3: Distribution centers of four species of *Xanthium* at different periods.
