# Supplementary material for: Global Patterns and Future Dynamics of Four Invasive Cocklebur Species Under Climate Change: Contrasting Climatic and Anthropogenic Drivers
Source: Biology (Basel). 2026 Mar 7;15(5):439. doi: 10.3390/biology15050439 (PMC12985310; doi:10.3390/biology15050439)
Supplement: Supplementary file 1 [file biology-15-00439-s001.zip › biology-4139446-supplementary.pdf]

Supplementary Information to the paper:

Table S1 Initially selected environment variables

Variables	Description	Unit
bio1	Annual Mean Temperature	℃
bio2	Mean Diurnal Range	℃
bio3	Isothermally	%
bio4	Temperature Seasonality	Standard Deviation ×100
bio5	Max Temperature of Warmest Month	℃
bio6	Min Temperature of Coldest Month	℃
bio7	Annual Range of Temperature	℃
bio8	Mean Temperature of Wettest Quarter	℃
bio9	Mean Temperature of Driest Quarter	℃
bio10	Mean Temperature of Warmest Quarter	℃
bio11	Mean Temperature of Coldest Quarter	℃
bio12	Annual Precipitation	mm
bio13	Precipitation of Wettest Month	mm
bio14	Precipitation of Driest Month	mm
bio15	Precipitation Seasonality	Coefficient of Variation
bio16	Precipitation of Wettest Quarter	mm
bio17	Precipitation of Driest Quarter	mm
bio18	Mean Precipitation of Warmest Quarter	mm
bio19	Mean Precipitation of Coldest Quarter	mm
t_carbon	Topsoil Organic Carbon	% weight
t_sand	Topsoil Sand Fraction	% weight
t_ph	Topsoil pH	-log(H^+)
t_root	Topsoil root depth	mm
t_tn	Topsoil total nitrogen content	% weight
pop	Population	population /km^2
GDP	Gross Domestic Product	USD
Land	Land-use Type	—
altitude	Altitude	m
slope	Slope	°
aspect	Aspect	°

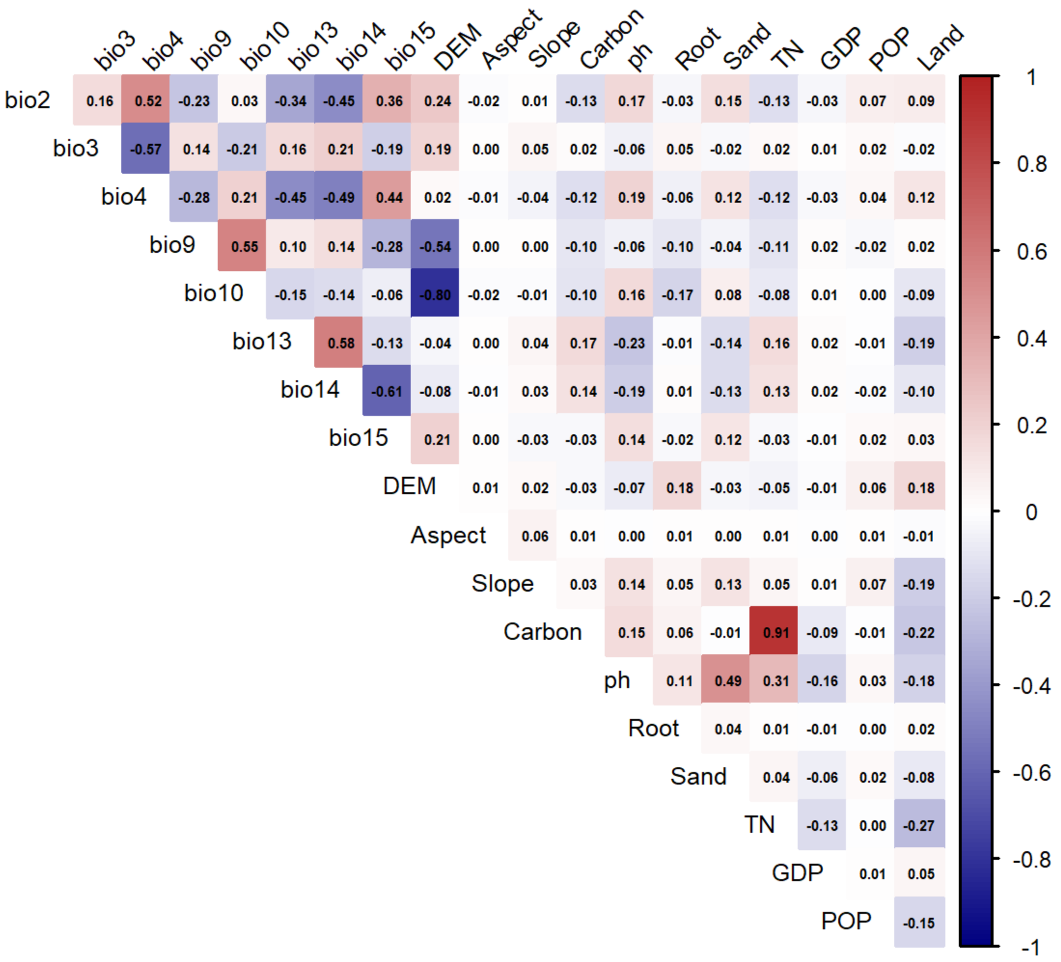

Figure S1 Pearson correlation analysis of various environment variables (r < 0.75).

Table S2 Model accuracy of four kinds at different periods

Species	Period	Scenarios	AUC	TSS
Cyclachaena xanthiifolia	Present	-	0.9474	0.8212
	2030s	SSP126	0.9488	0.8214
		SSP245	0.9475	0.8169
		SSP585	0.9469	0.8125
	2050s	SSP126	0.9496	0.8420
		SSP245	0.9492	0.8259
		SSP585	0.9495	0.8435
	2070s	SSP126	0.9490	0.8435
		SSP245	0.9494	0.8293
		SSP585	0.9496	0.8516
Xanthium chinense	Present	-	0.9950	0.9376
	2030s	SSP126	0.9965	0.9610
		SSP245	0.9964	0.9483
		SSP585	0.9968	0.9562
	2050s	SSP126	0.9965	0.9347
		SSP245	0.9966	0.9404
		SSP585	0.9965	0.9681
	2070s	SSP126	0.9958	0.9552
		SSP245	0.9970	0.9708
		SSP585	0.9965	0.9470
Xanthium italicum	Present	-	0.9794	0.8631
	2030s	SSP126	0.9809	0.8711
		SSP245	0.9803	0.8610
		SSP585	0.9803	0.8596
	2050s	SSP126	0.9797	0.8702
		SSP245	0.9806	0.8738
		SSP585	0.9814	0.9091
	2070s	SSP126	0.9813	0.8889
		SSP245	0.9807	0.8923
		SSP585	0.9821	0.8886
Xanthium spinosum	Present	-	0.8800	0.6603
	2030s	SSP126	0.8810	0.6627
		SSP245	0.8803	0.6548
		SSP585	0.8816	0.6622
	2050s	SSP126	0.8813	0.6687

Species	Period	Scenarios	AUC	TSS
		SSP245	0.8823	0.6687
		SSP585	0.8808	0.7275
		SSP126	0.8833	0.7296
	2070s	SSP245	0.8837	0.7382
		SSP585	0.8812	0.7349

Table S3 Distribution centers of four species of *Xanthium* at different periods

Species	Continent	Period	Scenarios	Longitude (°)	Latitude (°)	Altitude (m)
Cyclachaena xanthiifolia	Asia	Present	-	70.073102	48.107032	379
		2030s	SSP126	72.092648	49.165480	527
			SSP245	71.812657	49.186611	504
			SSP585	71.001620	49.159663	517
		2050s	SSP126	70.994250	49.690431	402
			SSP245	70.880659	49.733822	401
			SSP585	69.503939	49.127115	429
		2070s	SSP126	70.273783	48.909769	477
			SSP245	70.100832	48.914613	455
			SSP585	72.731418	48.592079	718
	Europe	Present	-	32.005102	52.449710	165
		2030s	SSP126	33.019151	53.049148	196
			SSP245	32.934023	53.238717	185
			SSP585	34.049288	53.180127	195
		2050s	SSP126	34.029504	53.217475	200
			SSP245	32.944462	53.153470	204
			SSP585	32.948336	52.905909	213
		2070s	SSP126	32.573933	53.070185	141
			SSP245	32.199843	52.493286	169
			SSP585	33.566096	52.699850	192
	North America	Present	-	-102.892027	44.065994	921
		2030s	SSP126	-103.640092	43.851317	1765
			SSP245	-104.412004	44.794715	1193
			SSP585	-104.710624	44.133631	1353
		2050s	SSP126	-105.468888	44.354631	1327
			SSP245	-104.745295	44.190901	1297
			SSP585	-102.484661	45.081294	800
		2070s	SSP126	-104.501779	44.160983	1385
			SSP245	-104.270682	44.383829	1410
			SSP585	-104.854822	44.687216	1243
Xanthium chinense	Asia	Present	-	114.863742	32.674079	39
		2030s	SSP126	116.125463	33.402094	31
			SSP245	115.909806	33.193134	30
			SSP585	117.297334	33.722973	22
		2050s	SSP126	115.291339	32.138582	46
			SSP245	115.949954	33.845916	37
			SSP585	116.464433	33.636458	30
		2070s	SSP126	114.899284	33.179447	42
			SSP245	115.199558	32.1669	41
			SSP585	114.894365	32.677283	40
	North America	Present	-	-84.028736	37.624594	361
		2030s	SSP126	-82.088769	37.569569	342
			SSP245	-83.211842	37.952949	243
			SSP585	-83.117535	38.372033	313
		2050s	SSP126	-83.427846	38.287192	311
			SSP245	-82.857777	38.478298	184

Species	Continent	Period	Scenarios	Longitude (°)	Latitude (°)	Altitude (m)
			SSP585	-82.49026	37.52358	356
		2070s	SSP126	-82.449286	37.661917	283
			SSP245	-81.834661	36.905381	826
			SSP585	-80.327357	38.459926	841
Xanthium italicum	Asia	Present	-	98.297449	36.595619	3257
		2030s	SSP126	98.08988	35.26182	4710
			SSP245	98.58598	35.378228	4311
			SSP585	97.623394	35.530914	4031
		2050s	SSP126	99.915667	35.522431	3105
			SSP245	97.125314	35.93608	4522
			SSP585	96.447497	35.430254	4361
		2070s	SSP126	98.617033	36.257315	4104
			SSP245	98.03296	35.01267	4234
			SSP585	99.807623	35.388034	4763
	Europe	Present	-	18.112031	48.471001	150
		2030s	SSP126	18.744479	48.638809	588
			SSP245	14.901016	47.867998	1048
			SSP585	16.684728	48.198995	147
		2050s	SSP126	15.575782	48.236332	324
			SSP245	15.309249	48.535567	657
			SSP585	15.494282	47.572505	721
		2070s	SSP126	14.901798	47.768135	811
			SSP245	15.499524	47.697814	1332
			SSP585	15.772767	47.511101	877
	North America	Present	-	-89.212484	39.696544	183
		2030s	SSP126	-92.929125	40.356703	319
			SSP245	-93.230411	39.605888	206
			SSP585	-93.62452	39.977077	222
		2050s	SSP126	-93.225715	40.090501	243
			SSP245	-93.882203	40.446084	302
			SSP585	-91.842476	39.839442	185
		2070s	SSP126	-91.515155	40.190309	147
			SSP245	-91.658362	39.24411	234
			SSP585	-91.488509	39.259496	235
	South America	Present	-	-61.567021	-35.419521	85
		2030s	SSP126	-61.901874	-34.363557	98
			SSP245	-61.709233	-33.496734	106
			SSP585	-61.846504	-34.533015	99
		2050s	SSP126	-62.254809	-35.120414	104
			SSP245	-62.554007	-35.563081	89
			SSP585	-62.323119	-34.886585	108
		2070s	SSP126	-61.699444	-34.734394	90
			SSP245	-61.133934	-33.501172	92
			SSP585	-62.323584	-35.11417	101
Xanthium spinosum	Africa	Present	-	22.586582	0.249611	453
		2030s	SSP126	23.232317	-0.734979	435
			SSP245	23.223797	0.101127	565

Species	Continent	Period	Scenarios	Longitude (°)	Latitude (°)	Altitude (m)
			SSP585	23.560194	2.264208	518
			SSP126	23.781291	-1.185527	451
		2050s	SSP245	22.851987	2.139088	381
			SSP585	23.339094	-0.952611	424
			SSP126	23.762701	0.4361	458
		2070s	SSP245	22.135831	2.290505	348
			SSP585	22.844355	-0.245877	533
		Present	-	70.927413	35.733031	4659
			SSP126	71.302224	35.69491	3456
		2030s	SSP245	68.227913	34.642363	3250
			SSP585	67.381216	36.678129	748
			SSP126	65.499392	36.6865	411
	Asia	2050s	SSP245	67.9884	36.846763	357
			SSP585	68.18314	36.21618	1161
			SSP126	67.095364	36.731116	346
		2070s	SSP245	67.710934	36.35409	1246
			SSP585	68.204508	36.9028	342
		Present	-	17.250127	50.11321	898
			SSP126	18.716451	49.817857	274
		2030s	SSP245	18.690858	50.279347	235
			SSP585	18.449382	50.152637	234
			SSP126	18.953116	50.072794	262
	Europe	2050s	SSP245	18.042113	50.046969	224
			SSP585	18.397586	50.136606	195
			SSP126	18.150554	49.458508	394
		2070s	SSP245	18.548801	49.74723	344
Xanthium spinosum			SSP585	19.898255	50.044725	441
		Present	-	-104.95198	37.705588	1968
			SSP126	-103.777422	37.574776	1527
		2030s	SSP245	-104.559551	37.661218	1879
			SSP585	-105.400684	38.373465	2452
			SSP126	-104.600434	38.100937	1580
	North America	2050s	SSP245	-105.38045	37.886233	2607
			SSP585	-104.710257	37.384586	2144
			SSP126	-104.014071	37.574254	1607
		2070s	SSP245	-105.118671	38.252591	1905
			SSP585	-102.514208	37.064921	1291
		Present	-	141.941992	-31.946192	158
			SSP126	143.62505	-32.182297	79
		2030s	SSP245	143.939942	-32.335404	88
			SSP585	143.76748	-32.19671	86
			SSP126	144.247223	-32.39788	91
	Oceania	2050s	SSP245	143.994788	-32.306167	85
			SSP585	144.886297	-32.63701	99
			SSP126	144.101247	-32.285102	88
		2070s	SSP245	144.015241	-32.361459	87
			SSP585	143.452535	-31.971615	77

Species	Continent	Period	Scenarios	Longitude （°）	Latitude （°）	Altitude （m）
		Present	-	-63.320329	-31.175484	164
			SSP126	-63.846638	-32.471653	295
		2030s	SSP245	-64.042275	-32.521409	409
			SSP585	-63.852575	-31.127172	316
	South America		SSP126	-63.954837	-31.974656	352
		2050s	SSP245	-64.598159	-32.195438	655
			SSP585	-64.689246	-32.333468	998
			SSP126	-64.294448	-32.069156	526
		2070s	SSP245	-64.922549	-32.6878	1476
			SSP585	-63.812884	-32.6216	283

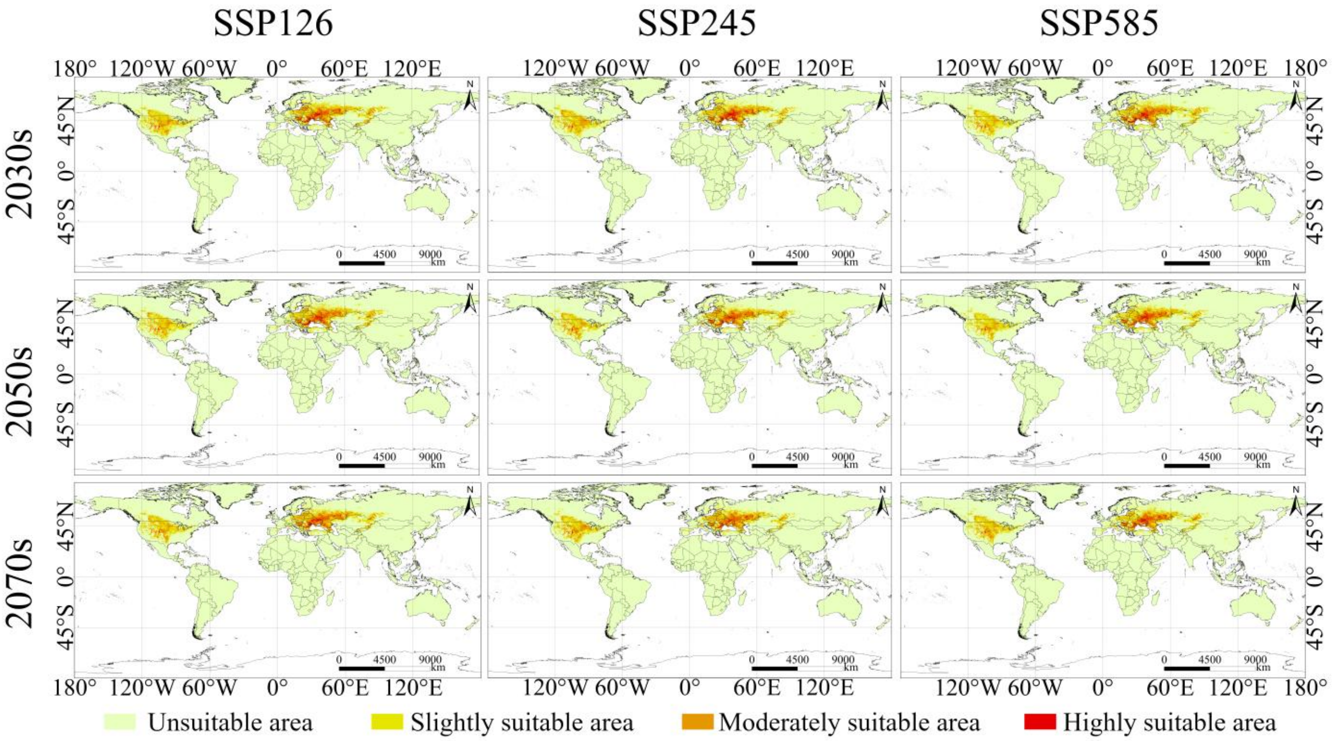

Figure S2 Predicted suitable areas of *Cyclachaena xanthiifolia* under different climate change scenarios.

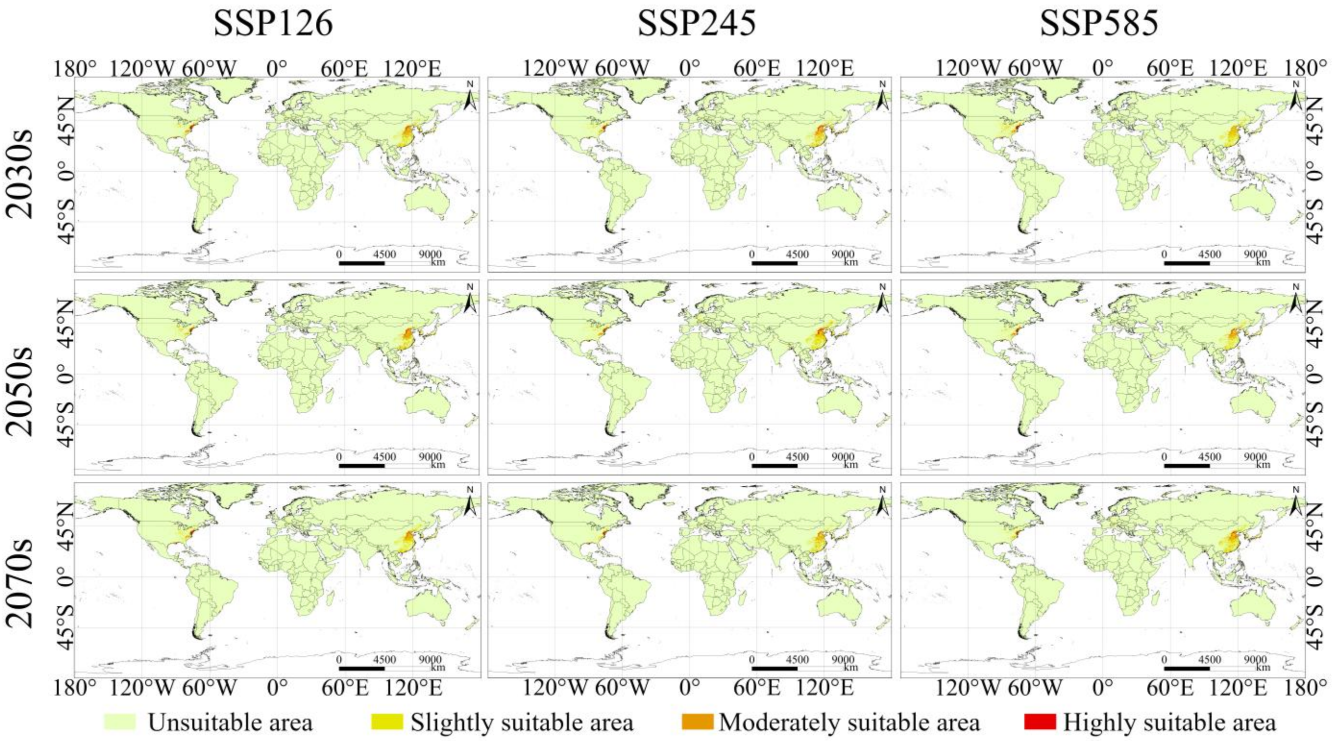

Figure S3 Predicted suitable areas of *Xanthium chinense* under different climate change scenarios.

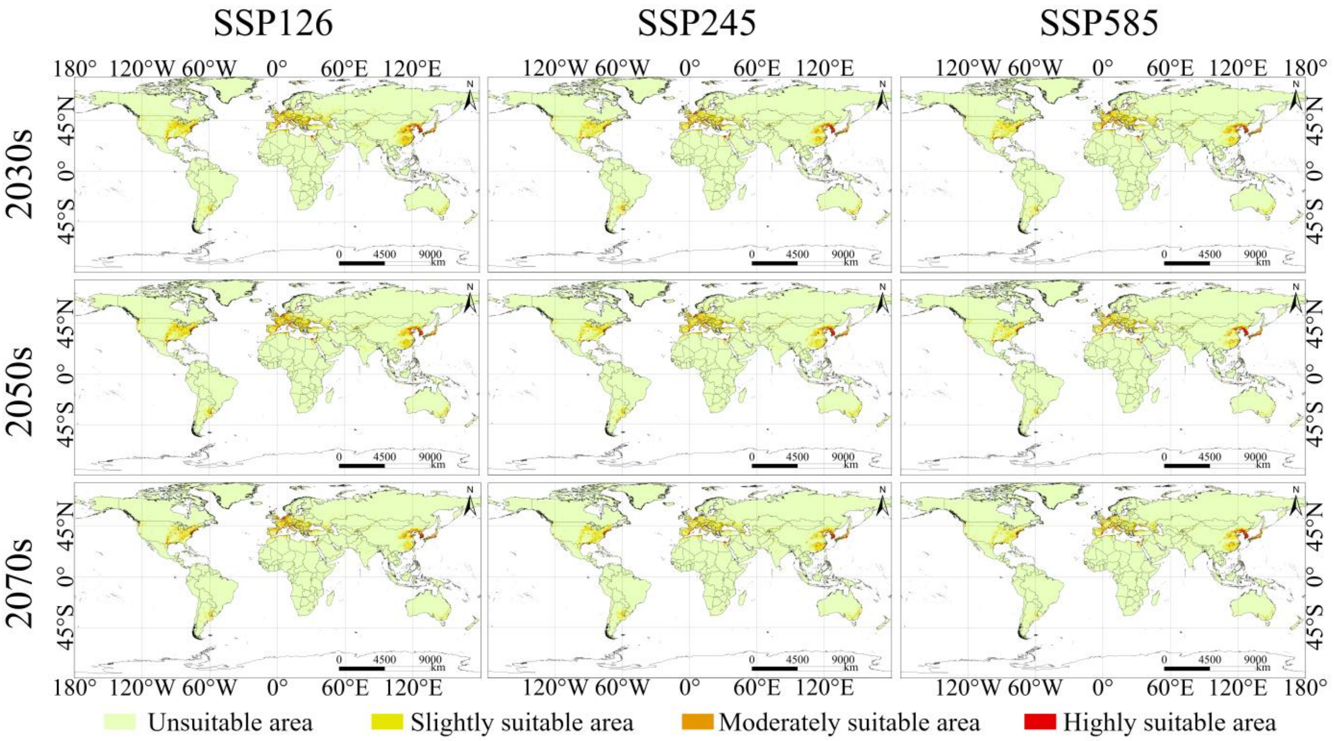

Figure S4 Predicted suitable areas of *Xanthium italicum* under different climate change scenarios.

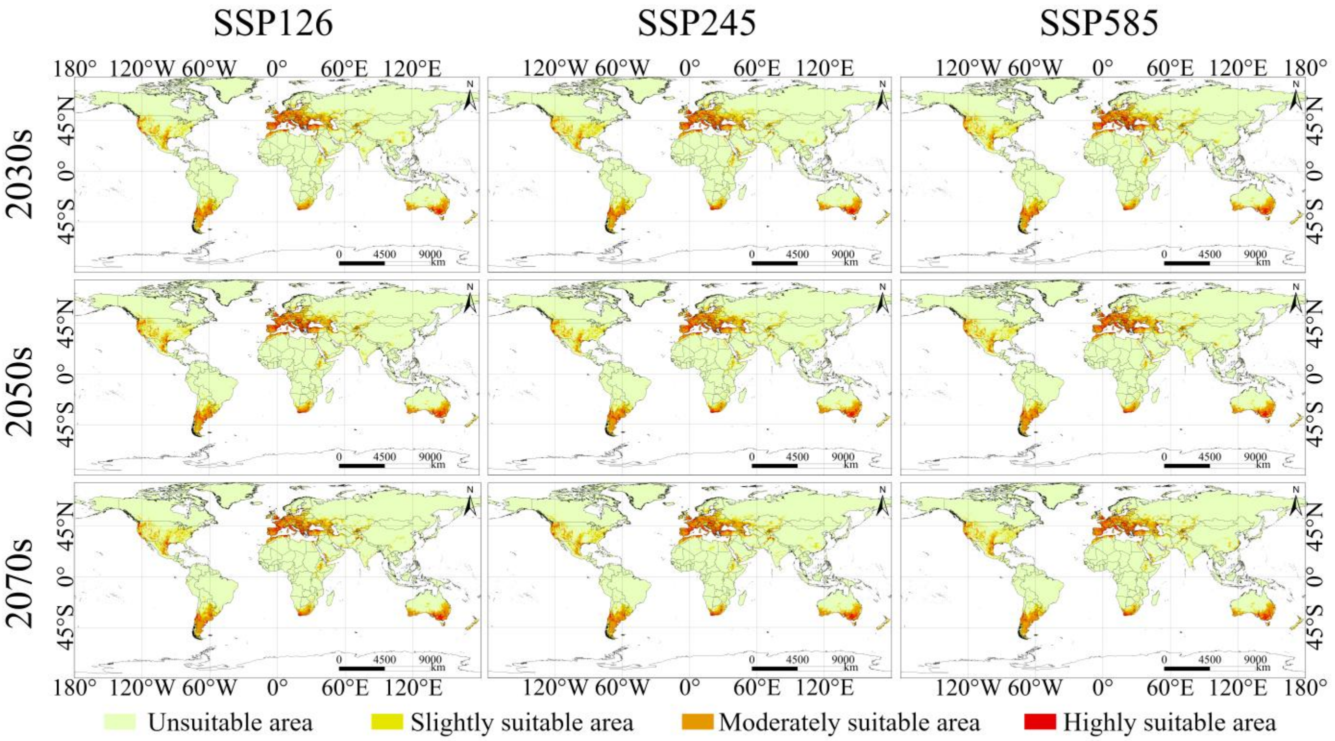

Figure S5 Predicted suitable areas of *Xanthium spinosum* under different climate change scenarios.

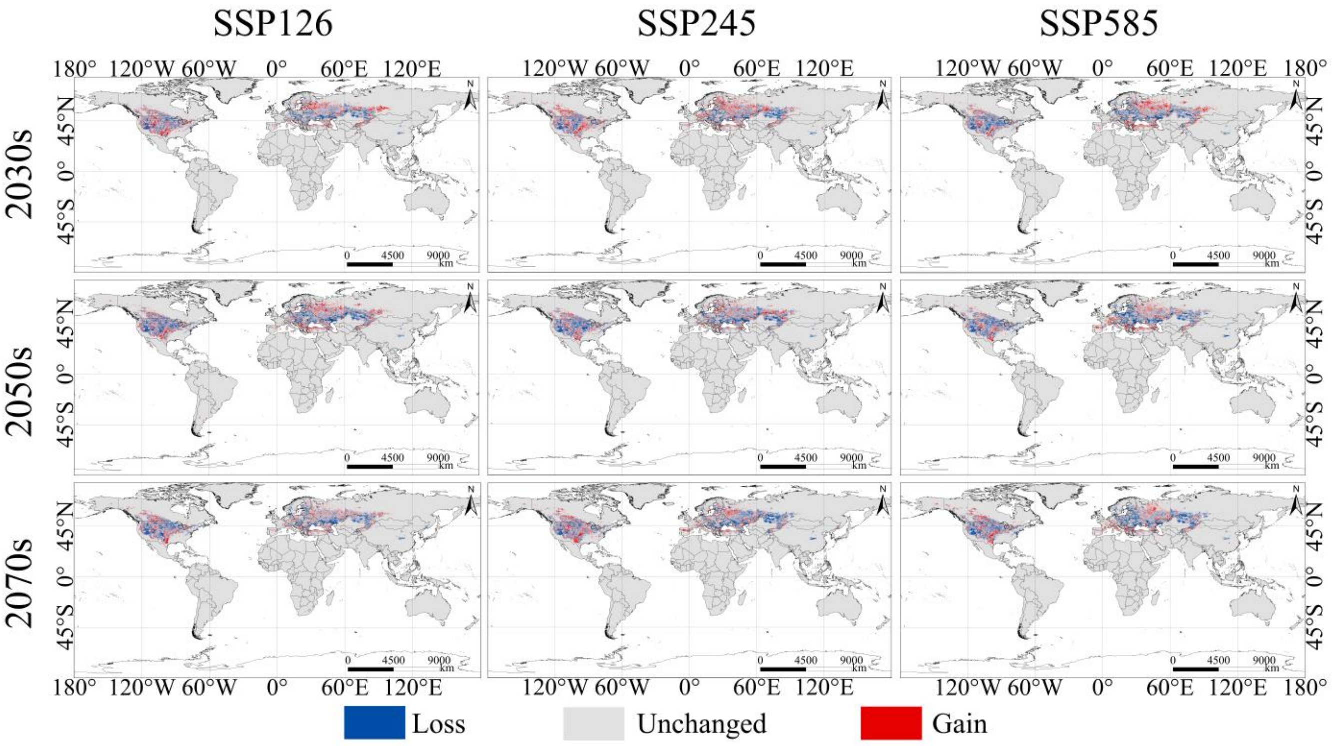

Figure S6 Spatial distribution changes of *Cyclachaena xanthiifolia* under different climate change scenarios.

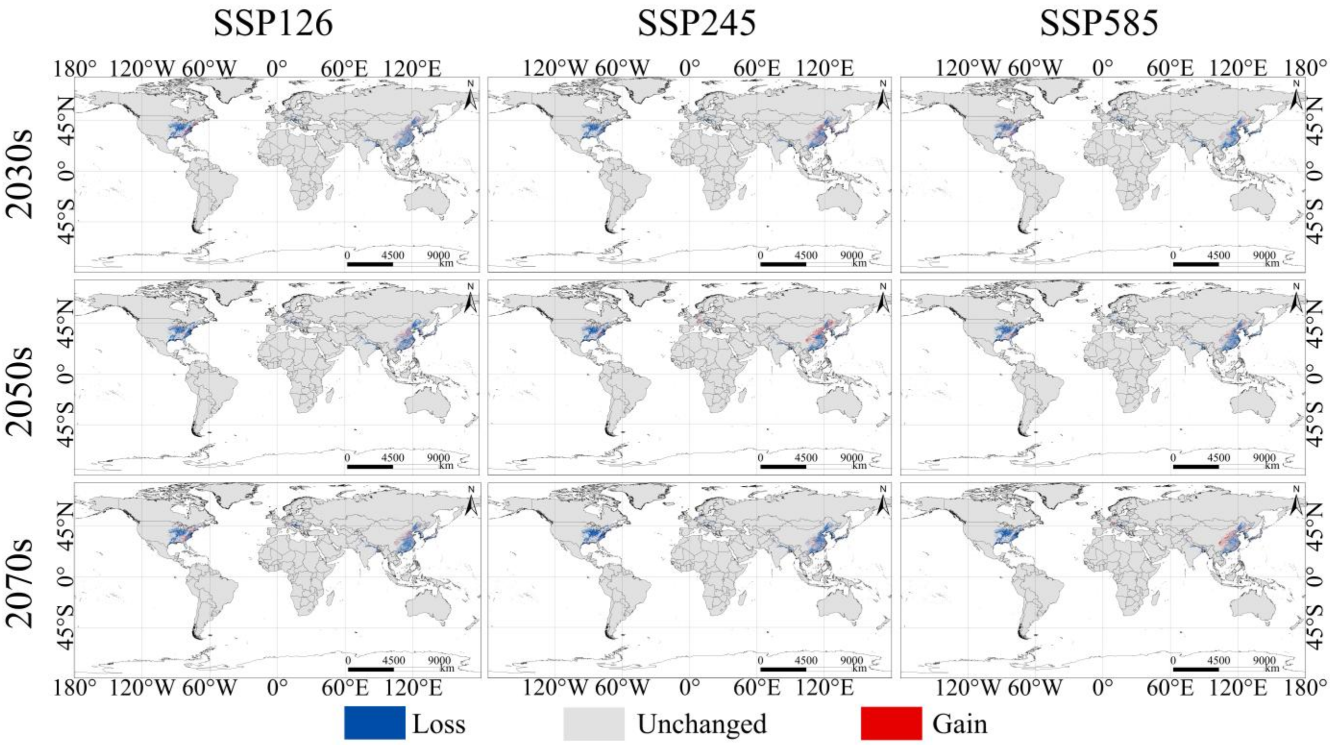

Figure S7 Spatial distribution changes of *Xanthium chinense* under different climate change scenarios.

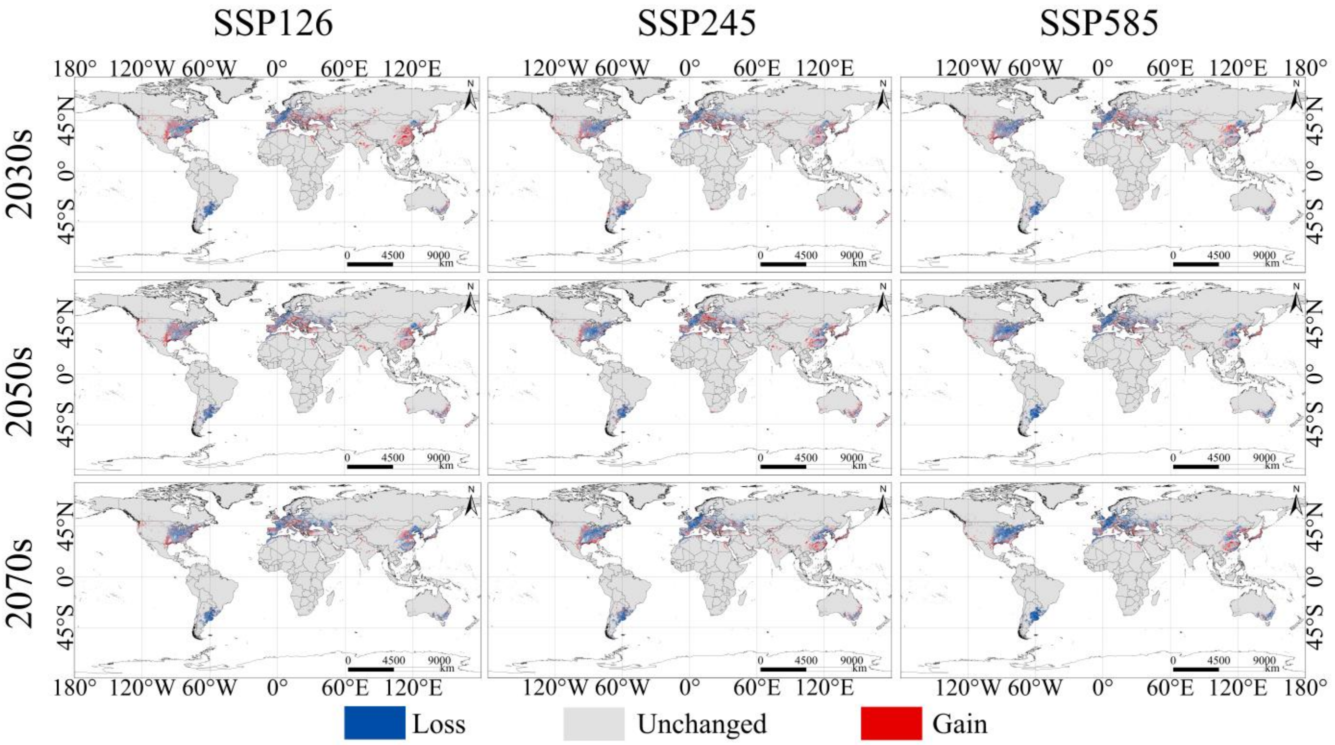

Figure S8 Spatial distribution changes of *Xanthium italicum* under different climate change scenarios.

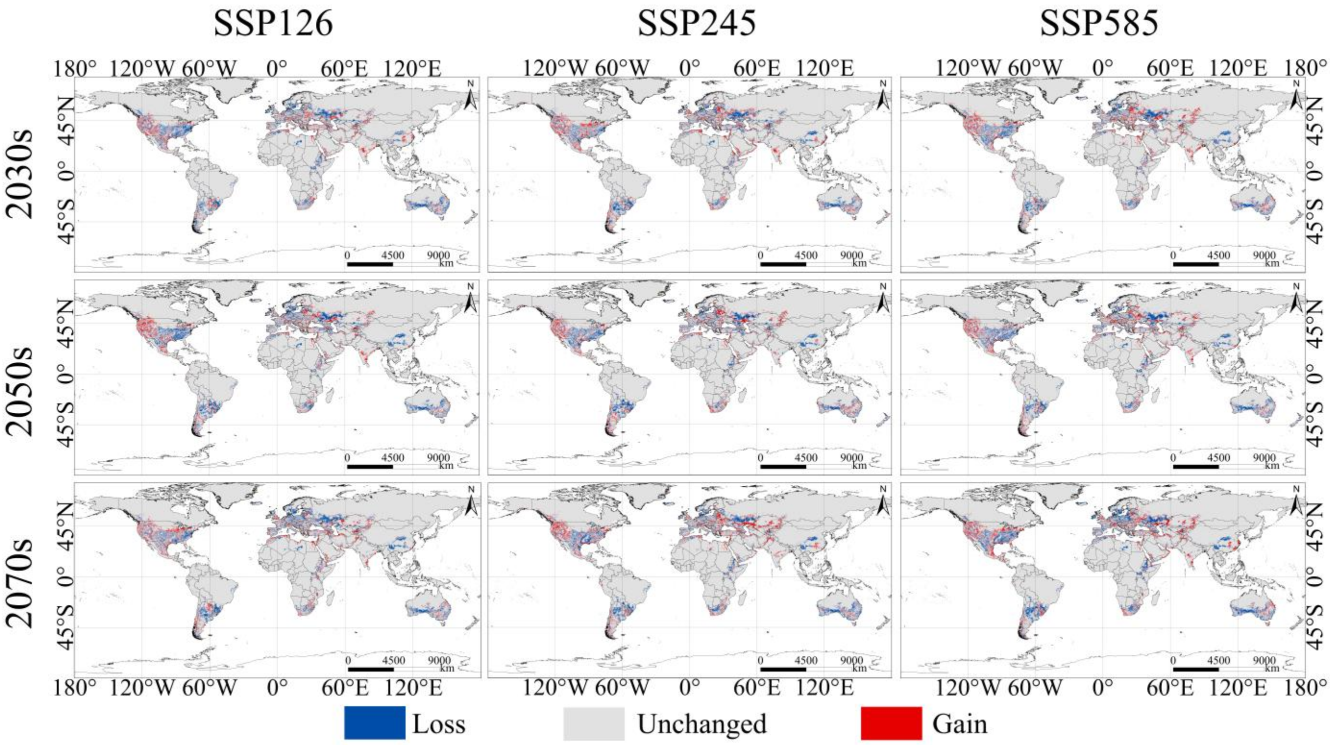

Figure S9 Spatial distribution changes of *Xanthium spinosum* under different climate change scenarios.

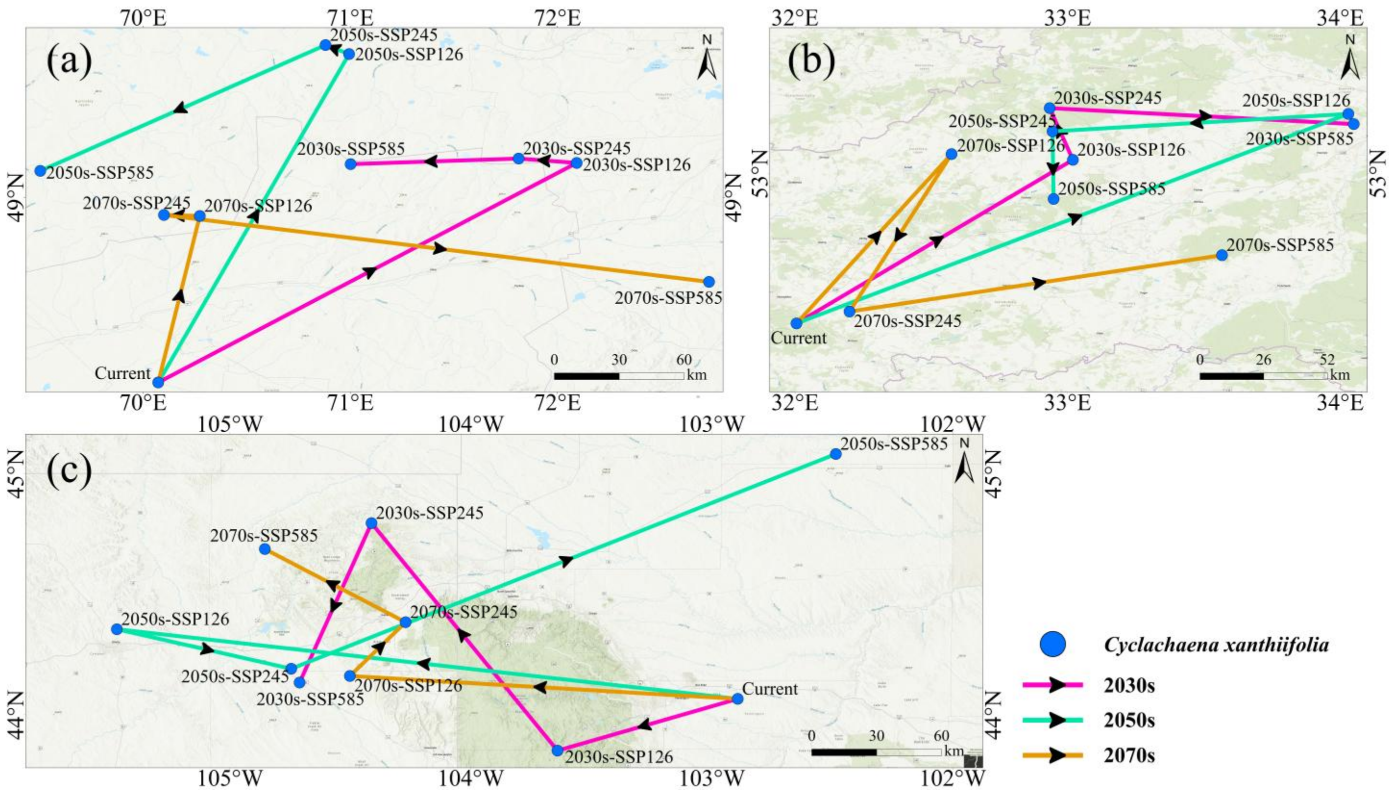

Figure S10 Migration trajectories of the distribution centers of *Cyclachaena xanthiifolia* in different continents (a) Asia, (b) Europe, (c) North America under different climate change scenarios.

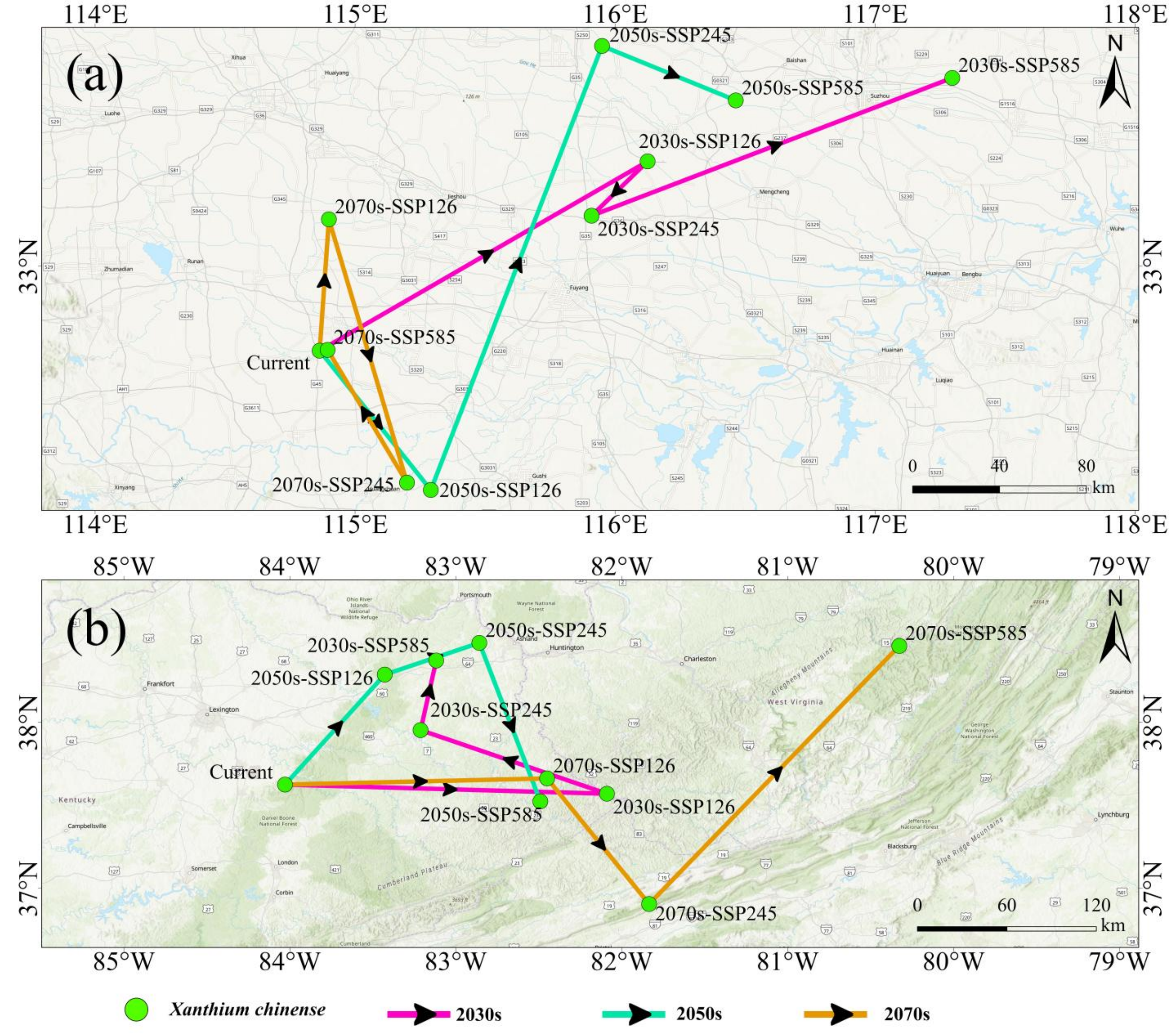

Figure S11 Migration trajectories of the distribution centers of *Xanthium chinense* in different continents (a) Asia, (b) North America under different climate change scenarios.

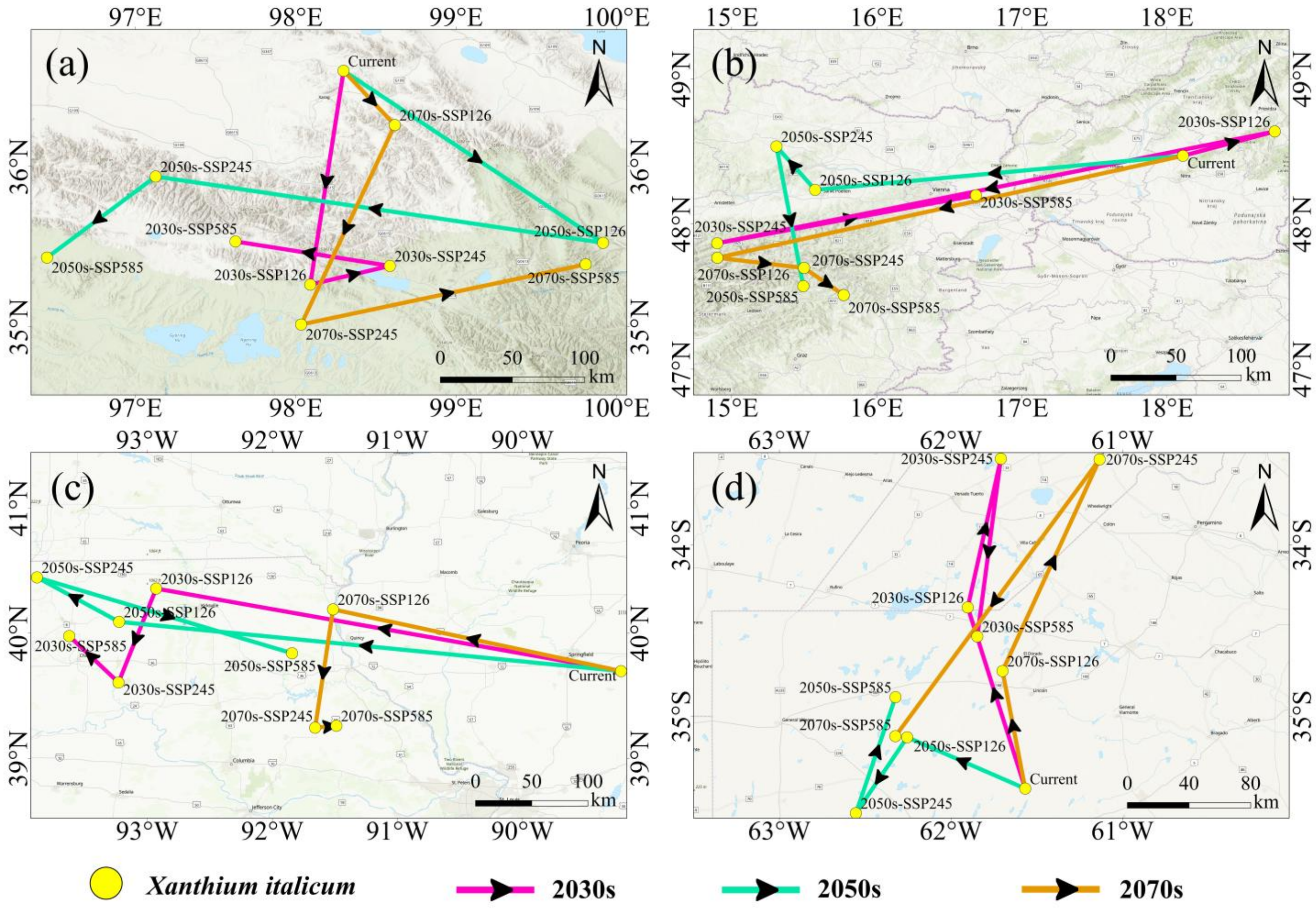

Figure S12 Migration trajectories of the distribution centers of *Xanthium italicum* in different continents (a) Asia, (b) Europe, (c) North America, (d) South America under different climate change scenarios.

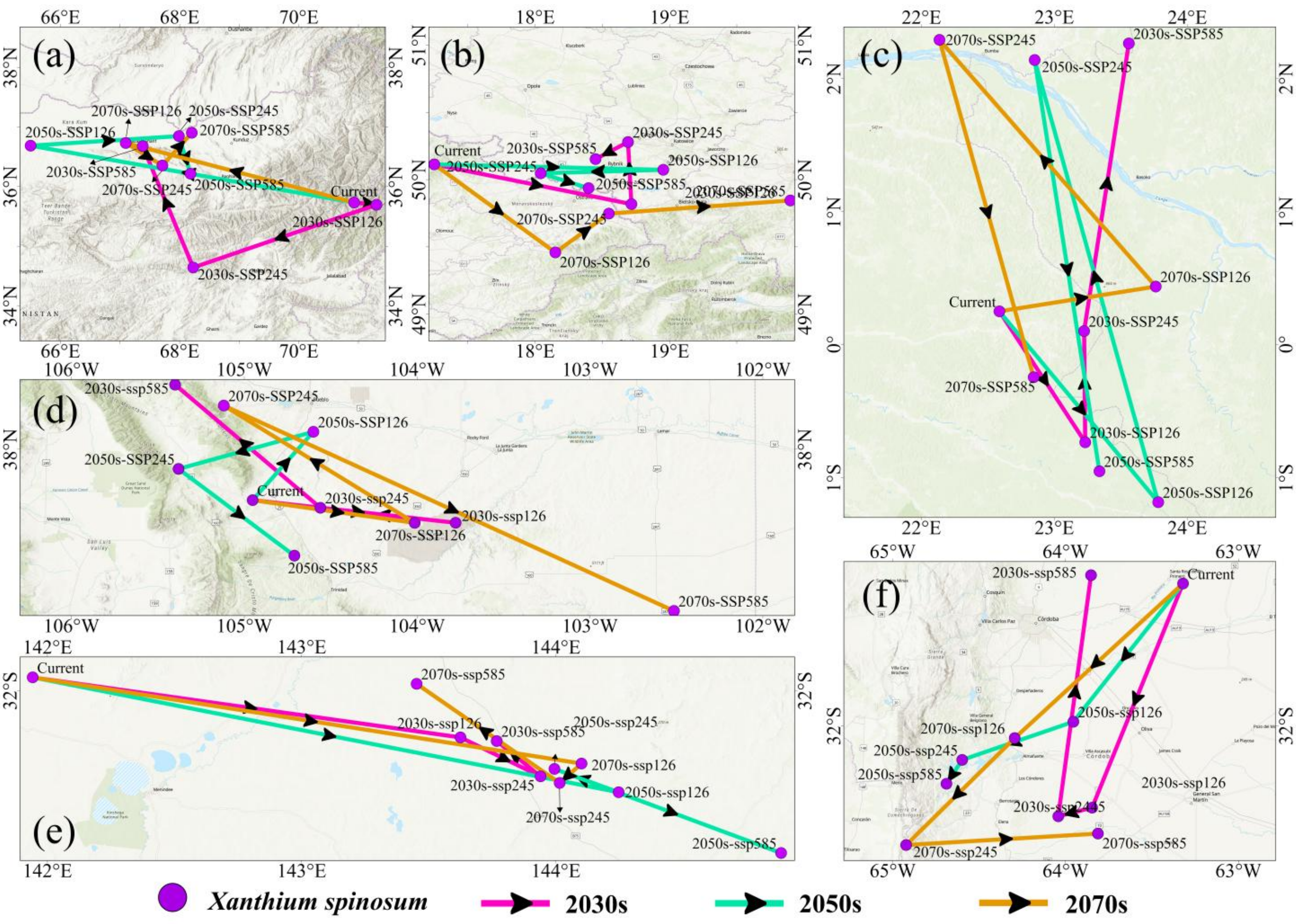

Figure S13 Migration trajectories of the distribution centers of *Xanthium spinosum* in different continents (a) Asia, (b) Europe, (c) Africa, (d) North America, (e) Oceania, (f) South America under different climate change scenarios.
